# CodY Regulates the Activity of the Virulence Quorum Sensor PlcR by Controlling the Import of the Signaling Peptide PapR in *Bacillus thuringiensis*

**DOI:** 10.3389/fmicb.2015.01501

**Published:** 2016-01-06

**Authors:** Leyla Slamti, Christelle Lemy, Céline Henry, Alain Guillot, Eugénie Huillet, Didier Lereclus

**Affiliations:** Micalis Institute, INRA, AgroParisTech, Université Paris-SaclayJouy-en-Josas, France

**Keywords:** quorum-sensing, regulation, signaling peptide, Opp, virulence regulon, mass spectrometry, shot gun

## Abstract

In Gram-positive bacteria, cell–cell communication mainly relies on cytoplasmic sensors of the RNPP family. Activity of these regulators depends on their binding to secreted signaling peptides that are imported into the cell. These quorum sensing regulators control important biological functions in bacteria of the *Bacillus cereus* group, such as virulence and necrotrophism. The RNPP quorum sensor PlcR, in complex with its cognate signaling peptide PapR, is the main regulator of virulence in *B. cereus* and *Bacillus thuringiensis* (Bt). Recent reports have shown that the global stationary phase regulator CodY, involved in adaptation to nutritional limitation, is required for the expression of virulence genes belonging to the PlcR regulon. However, the mechanism underlying this regulation was not described. Using genetics and proteomics approaches, we showed that CodY regulates the expression of the virulence genes through the import of PapR. We report that CodY positively controls the production of the proteins that compose the oligopeptide permease OppABCDF, and of several other Opp-like proteins. It was previously shown that the pore components of this oligopeptide permease, OppBCDF, were required for the import of PapR. However, the role of OppA, the substrate-binding protein (SBP), was not investigated. Here, we demonstrated that OppA is not the only SBP involved in the recognition of PapR, and that several other OppA-like proteins can allow the import of this peptide. Altogether, these data complete our model of quorum sensing during the lifecycle of Bt and indicate that RNPPs integrate environmental conditions, as well as cell density, to coordinate the behavior of the bacteria throughout growth.

## Introduction

Quorum-sensing (QS) is a mode of cell–cell communication that controls important adaptive processes in bacteria such as conjugation, virulence, sporulation, or competence (Dunny and Leonard, 1997; Perego, 2013; Cook and Federle, 2014; Slamti et al., 2014). This type of communication depends on the secretion of small molecules that trigger regulation mechanisms. In Gram-positive species QS mainly relies on the secretion of auto-inducing oligopeptides. These autoinducers can either act on the outside of the bacterium, by interacting with a sensor in the membrane, or in the cytoplasm of the cell. In the latter case, once internalized, the peptides bind an effector protein whose activity is subsequently modified (Perego and Hoch, 1996; Dunny and Leonard, 1997; Lazazzera et al., 1997; Miller and Bassler, 2001). Examples of these interactions are provided by the protein-peptide pairs of the RNPP family (for Rap-NprR-PrgX-PlcR, the defining members of this family; Declerck et al., 2007). The Rap phosphatases-Phr peptides are involved in competence and sporulation in *Bacillus subtilis* (Lazazzera et al., 1997), the PrgX transcriptional regulator-cCf10 peptide controls conjugation in *Enterococcus faecalis* (Suzuki et al., 1984) and NprR-NprX and PlcR-PapR, two transcriptional regulator/peptide pairs of the *Bacillus cereus* (Bc) group, are required for necrotrophism and virulence, respectively (Perchat et al., 2011; Dubois et al., 2012; Slamti et al., 2014).

The quorum-sensor PlcR is one of the defining proteins of the RNPP family and has been a model for understanding the mode of action of the protein-peptide complex at the molecular level (Grenha et al., 2013). PlcR is a transcription factor that regulates the expression of about 45 genes in the sporulating Gram-positive bacteria of the Bc group (Agaisse et al., 1999; Gohar et al., 2008). In addition to Bc *sensu stricto*, responsible for foodborne and opportunistic infections, this group comprises six other species including the causative agent of anthrax, *Bacillus anthracis* (Ba) and the insect pathogen *Bacillus thuringiensis* (Bt). PlcR is truncated and thus inactive in Ba (Agaisse et al., 1999; Mignot et al., 2001). However, the opportunistic properties of Bc and Bt (i.e., the ability to cause non-foodborne infections) are PlcR-dependent (Raymond et al., 2010). Most of the PlcR-controlled genes in Bc and Bt encode proteins whose functions are related to food supply and virulence (phospholipases, proteases, hemolysins, and toxins), cell protection and environment-sensing. Deletion of *plcR* results in a drastic reduction of Bt and Bc virulence in insect and mouse models of infection (Salamitou et al., 2000).

Activity of the regulator depends on the binding of the signaling peptide PapR (Slamti and Lereclus, 2002), which was shown to be imported by the oligopeptide permease Opp (Gominet et al., 2001). Oligopeptide permeases are ATP-binding cassette transporters composed of five proteins: two integral membrane proteins that form the actual pore (OppB and OppC), two ATPases bound to the membrane proteins that provide the energy required for translocation (OppD and OppF) and a membrane-anchored substrate-binding protein (SBP) facing the outside of the cell (OppA; Hiles et al., 1987; Higgins, 1992; Hiron, 2007). While it was reported that OppB-C-D-F that form the pore of the permease were required for the import of PapR (Gominet et al., 2001), the involvement of OppA, was not described. Regulation of the PlcR-PapR system is quite complex as PlcR activates its own transcription and that of the genes under its control, which include *papR*, at the end of the exponential growth phase (Lereclus et al., 1996; Agaisse et al., 1999). In addition, the master regulator of sporulation, Spo0A, represses the transcription of *plcR* during stationary phase by binding and presumably obstructing the *plcR* promoter region (Lereclus et al., 2000; Gominet et al., 2001). It has also been recently reported that the global regulator CodY was required for expression of *plcR* and PlcR-dependent genes in Bc (Frenzel et al., 2012; Lindback et al., 2012). However, the influence of CodY on the expression of the virulence factors does not appear to be *via* a direct binding of CodY to the promoter regions of *plcR* or of the PlcR-dependent genes.

CodY is conserved in low G+C Gram-positive bacteria and is a transcriptional regulator of metabolism (Sonenshein, 2005, 2007). Activity of CodY depends on cofactors such as branched-chain amino acids and GTP, depending on the bacterial species. CodY mainly directly or indirectly represses metabolism-related genes during exponential growth. When the nutrient availability decreases, it induces a drop in the intracellular concentration of GTP and amino acids available for CodY activation. This leads to the derepression of CodY-dependent genes when cells enter stationary phase. Aside from controlling the direct response to starvation, CodY is also involved in the regulation of virulence genes in several pathogens (Sonenshein, 2005; Stenz et al., 2011; Richardson et al., 2015). CodY is required for toxin expression in Ba, *via* the control of AtxA levels in the cell. A Ba Δ*codY* deletion mutant is severely attenuated in its virulence against mice (van Schaik et al., 2009; Chateau et al., 2011). In *Clostridium difficile*, CodY represses toxin genes expression by direct binding to the promoter of *tcdR*, the sigma factor required for toxin gene transcription (Dineen et al., 2007), resulting in a hypervirulent phenotype for a Δ*codY* mutant (Richardson et al., 2015). In *Staphylococcus aureus*, the *codY* deletion leads to an increase of the hemolytic activity and increased virulence in a mouse model of infection (Montgomery et al., 2012; Rivera et al., 2012). This is due to the derepression of the QS Agr-dependent genes in exponential phase (Majerczyk et al., 2008) and a recent report showed that this CodY-mediated regulation was indirect (Roux et al., 2014). However, as for Bt and Bc, the authors did not report the mechanism responsible for the effect of CodY on the virulence regulators.

Here, we investigated the mechanism underlying this regulation. We report that CodY is involved in the expression of Bt virulence factors through its role in the import of the signaling peptide PapR. Using proteomics and genetics approaches, we show that a Δ*codY* mutant is impaired in its ability to import PapR. We also show that OppA is not the only SBP involved in the recognition of PapR, and that several other OppA-like proteins can allow the import of this peptide. This report brings new insights into the regulation of virulence gene expression and its link to the metabolic state of the bacteria.

## Materials and Methods

### Bacterial Strains and Growth Conditions

The acrystalliferous *B. thuringiensis* 407 Cry^-^ strain (Bt 407^-^; Lereclus et al., 1989) was used as the parental strain to create all the strains used in this study. *Escherichia coli* strain DH5α (Taylor et al., 1993) was used as the host strain for plasmid construction. *E. coli* strain ET12567 (MacNeil et al., 1992) was used to prepare DNA prior to electroporation in *B. thuringiensis*. Unless otherwise noted, cells were grown in LB medium (1% tryptone, 0.5% yeast extract, 1% NaCl) at 37°C and stored at -80°C in LB containing 15% glycerol.

For *B. thuringiensis* cultures, t0 corresponds to the beginning of the transition between the exponential and stationary growth phases. It is defined as the point where the slope starts to decrease at the end of the exponential phase.

Columbia agar plates (BioMérieux) containing 5% sheep blood were used to evaluate the hemolytic activity of the *B. thuringiensis* strains. BHI (Beckton-Dickinson) agar plates containing 5% egg yolk were used to evaluate the lecithinase activity of the *B. thuringiensis* strains.

The antibiotic concentrations used for selection of *B. thuringiensis* and *E. coli* were as follows: erythromycin, 10 μg/mL; kanamycin, 200 μg/mL; ampicillin, 100 μg/mL.

When required, xylose was used at a concentration of 20 mM.

### Strain Construction

The plasmids used for strain construction are listed in **Table 1**. A list of the strains used in this study is presented **Table 2**. Strain Bt ΔcodY was constructed as follows. pRN-ΔcodY was integrated in the chromosome of Bt 407^-^ following a single recombination event as described previously (Lereclus et al., 1992) and verified by PCR using primer pairs codY7/RN3 and RN2/codYVerifRev (Supplementary Table S1). The resulting Bt *codY*::pRN-Δ*codY* cells were then transformed with p1618K-P*xyl* or p1618K-P*xyl*’-*codY*. The second recombination event was allowed to proceed in these strains in parallel with the parental Bt *codY*::pRN-Δ*codY* cells. Occurrence of this event was screened by PCR using primer pair codY7/codYDnBam (Supplementary Table S1) on the chromosome of Erm^S^ (and Kan^R^ for the strains harboring the replicative plasmid) cells. Supplementary Table S2 shows the number of clones tested to screen for *codY* deletion. We could only obtain a deletion of the *codY* gene in cells harboring a copy of *codY* in *trans*. We then cultured Bt Δ*codY* (p1618K-P*xyl*’-*codY*) cells in parallel with the Bt (p1618K-P*xyl*’-*codY*) strain and compared the percentage of cells that lost the plasmid in these two strains (Supplementary Materials and Methods). Supplementary Table S3 shows the number of clones tested. Only 10% of the Bt ΔcodY cells had lost the plasmid at the end of the experiment. In contrast, there was a marked loss of the plasmid in wild-type Bt cells. We chose one of the Bt ΔcodY mutants to pursue our experiments. In order to complement the mutation this clone was transformed with (p1618K-P*xyl*’-*codY*) resulting in strain Bt Δ*codY* (p1618K-P*xyl*’-*codY*)_B_.

**Table 1:** Plasmids used this study.

Name	Relevant features	Reference
pRN-Δ*codY*	*codY* flanking regions were amplified by PCR from the chromosome of Bt 407^-^ using primer pairs codYUp-HindIII/codYUp-XbaI and codYDn-XbaI/codYDn-BamHI and cloned between the *Bam*HI and *Hin*dIII restriction sites of the thermosensitive plasmid pRN5101 (Villafane et al., 1987) to generate a 504 bp internal deletion in *codY*	This study
p1618K-P*xyl*	Replicative multicopy vector harboring the xylose-inducible promoter of *xylA*	Dubois et al., 2013
p1618K-P*xyl*’-*codY*	Transcription of *codY* is driven by the xylose-inducible promoter of *xylA*	Dubois et al., 2013
p304-P*xyl*+	P*xylA* was amplified by PCR from the chromosome of *Bacillus subtilis* strain 168 using primer pair Pxyl1/PxylRBS+ and cloned between the *Sph*I and *Xba*I sites of the replicative multicopy pHT304 plasmid (Arantes and Lereclus, 1991). p304-P*xyl*+ harbors a modified version of the xylose-inducible promoter region of *xylA* to enhance translation efficiency (Stammen et al., 2010)	This study
p304-P*xyl*+’-*plcR*	*plcR* was amplified by PCR from the chromosome of strain Bt 407^-^ using primer pair SP1/PO2 and cloned between the *Nco*I and *Bam*HI restriction sites of p304-P*xyl*+	This study
p304-P*xyl*+’-*papR*_7i_	Primers PapR7i1 and PapR7i2 were annealed as previously described (Slamti and Lereclus, 2002) and the resulting double-stranded DNA fragment encoding the ADLPFEF peptide was cloned between the *Nco*I and *Eco*RI restriction sites of p304-P*xyl*+	This study
p304-P*xyl*+’-*papR*_FL_	*papR*_FL_ was amplified by PCR from the chromosome of strain Bt 407^-^ using primer pair PapRFL1/PapRFL2 and cloned between the *Nco*I and *Eco*RI restriction sites of p304-P*xyl*+	This study
pP*plcR*’-*Z*	The promoter region of *plcR* was amplified by PCR from the chromosome of strain Bt 407^-^ using primer pair Pp3/Pp2 and cloned between the *Hin*dIII and *Bam*HI restriction sites of the multicopy replicative vector pHT304.18-*lacZ* (Agaisse and Lereclus, 1994). The promoter of *plcR* drives the transcription of the reporter gene *lacZ*	This study
pP*plcA*’-*Z*	The promoter of *plcA* drives the transcription of *lacZ* in pHT304.18-*lacZ*	Lereclus et al., 1996
pP*papR*’-*Z*	The promoter of *papR* drives the transcription of *lacZ* in pHT304.18-*lacZ*	Agaisse et al., 1999
pMAD-Δ*oppA*	*oppA* flanking regions were amplified by PCR from the chromosome of Bt 407^-^ using primer pairs LSoppA1/LSoppA2 and LSoppA3/LSoppA4 followed by overlap extension PCR and cloned between the *EcoR*I and *BamH*I restriction sites of the thermosensitive plasmid pMAD (Arnaud et al., 2004)	This study
p304-P*xyl*+’-02170/06720/09790/12330/12390/20960/36620/36650/36660	The gene corresponding to each of the indicated locus tags was amplified by PCR from the chromosome of strain Bt 407^-^ using primer pair c02170.1/c02170.2, c06720.1/c06720.2, c09790.1/c09790.2, c12330.1/c12330.2, c12390.1/c12390.2, c20960.1/c20960.2, c36620.1/c36620.2, c36650.1/c36650.2 or c36660.1/c36660.2, and cloned between the *Nco*I and *Kpn*I restriction sites of p304-P*xyl*+	This study

**Table 2:** Strains used this study.

Name	Relevant features	Reference
Bt 407^-^	Acrystalliferous *Bacillus thuringiensis* strain 407	Lereclus et al., 1989
Bt ΔcodY (p1618K-P*xyl*’-*codY*)	Strain deleted for *codY* with a copy of the gene in *trans*	This study
Bt (p1618K-P*xyl*’-*codY*)	Wild-type strain with a copy of *codY* in *trans*	This study
Bt ΔcodY	Strain deleted for *codY*, obtained by curing the p1618K-P*xyl*’-*codY* plasmid from strain Bt ΔcodY (p1618K-P*xyl*’-*codY*)	This study
Bt ΔcodY (p1618K-P*xyl*’-*codY*)_B_	Bt ΔcodY transformed with p1618K-P*xyl*’-*codY* to complement the mutation	This study
Bt ΔcodY (pP*plcR*’-*Z*)	Bt ΔcodY in which we measure the activity of the promoter of *plcR*	This study
Bt ΔcodY (pP*plcA*’-*Z*)	Bt ΔcodY in which we measure the activity of the promoter of *plcA*	This study
Bt ΔcodY (pP*papR*’-*Z*)	Bt ΔcodY in which we measure the activity of the promoter of *papR*	This study
Bt (pP*plcR*’-*Z*)	Bt 407^-^ in which we measure the activity of the promoter of *plcR*	This study
Bt (pP*plcA*’-*Z*)	Bt 407^-^ in which we measure the activity of the promoter of *plcA*	This study
Bt (pP*papR*’-*Z*)	Bt 407^-^ in which we measure the activity of the promoter of *papR*	This study
Bt ΔcodY (p1618K-P*xyl*’-*codY*)_B_ (pP*plcA*’-*Z*)	Bt ΔcodY transformed with p1618K-P*xyl*’-*codY* to complement the mutation in which we measure the activity of the promoter of *plcA*	This study
Bt ΔcodY (p1618K-P*xyl*’) (pP*plcA*’-*Z*)	Bt ΔcodY transformed with p1618K-P*xyl*’ in which we measure the activity of the promoter of *plcA*	This study
Bt (p1618K-P*xyl*’) (pP*plcA*’-*Z*)	Bt 407^-^ transformed with p1618K-P*xyl*’ in which we measure the activity of the promoter of *plcA*	This study
Bt ΔcodY (p304-P*xyl*+’-*plcR*)	Bt ΔcodY in which we express *plcR* from the xylose-inducible promoter of *xylA*	This study
Bt ΔcodY (p304-P*xyl*+)	Bt ΔcodY carrying the empty p304-P*xyl*+ vector	This study
Bt (p304-P*xyl*+)	Bt 407^-^ carrying the empty p304-P*xyl*+ vector	This study
Bt ΔplcR	Strain Bt 407^-^ in which *plcR* has been replaced with the *aphA3* gene	Salamitou et al., 2000
Bt ΔplcR (p304-P*xyl*+’-*plcR*)	Bt ΔplcR in which we express *plcR* from the xylose-inducible promoter of *xylA*	This study
Bt ΔcodY (p304-P*xyl*+’-*papR*_7i_)	Bt ΔcodY in which we express *papR*_7i_ from the xylose-inducible promoter of *xylA*. The mature 7 aa form of PapR is produced directly inside the cells	This study
Bt ΔcodY (p304-P*xyl*+’-*papR*_FL_)	Bt ΔcodY in which we express *papR*_FL_ encoding the full-length form of PapR (including the signal sequence) from the xylose-inducible promoter of *xylA*	This study
Bt ΔpapR P*plcA*’-*lacZ*	Strain Bt 407^-^ in which *papR* has been replaced with the *aphA3* gene and carrying a chromosomal transcriptional fusion between the promoter region of *plcA* and the reporter gene *lacZ*	Slamti and Lereclus, 2002
Bt ΔpapR P*plcA*’-*lacZ* (p304-P*xyl*+’-*papR*_7i_)	Strain Bt ΔpapR P*plcA*’-*lacZ* in which we express *papR*_7i_ from the xylose-inducible promoter of *xylA*. The mature 7 aa form of PapR is produced directly inside the cells	This study
Bt ΔpapR P*plcA*’-*lacZ* (p304-P*xyl*+’-*papR*_FL_)	Strain Bt ΔpapR P*plcA*’-*lacZ* in which we express *papR*_FL_ encoding the full-length form of PapR (including the signal sequence) from the xylose-inducible promoter of *xylA*	This study
Bt ΔpapR	Strain Bt 407^-^ in which *papR* has been replaced with the *aphA3* gene using pRNΔ*papR*::Kan^R^ (Slamti and Lereclus, 2002)	This study
Bt ΔpapR (pP*plcA*’-*Z*)	Bt ΔpapR in which we measure the activity of the promoter of *plcA*	This study
Bt ΔoppA	Bt 407^-^ carrying a markerless deletion of *oppA* made with pMAD-Δ*oppA*	This study
Bt ΔcodY (p304-P*xyl*+’-02170/06720/09790/12330/12390/20960/36620/36650/36660)	Strains Bt ΔcodY in which we express each gene corresponding to the indicated locus tags from the xylose-inducible promoter of *xylA*	This study

### β-galactosidase Assay

β-galactosidase activities were measured as described previously with the exception of the incubation temperature which was set to 30°C instead of room temperature (Perchat et al., 2011). Each assay was repeated at least twice.

### Lecithinase Assay

Two mL samples were harvested from cell cultures by centrifugation at the indicated times for each experiment. The supernatant was then filter-sterilized and kept at 4°C overnight. 180 μL of a 0.5% egg yolk saline solution were distributed in a 96-well plate and 20 μL of each filtered supernatant was added to two wells. This was considered time zero of the assay. The plate was then incubated at 25°C in a Tecan Infinite F200 Pro reader for up to 20 h and the OD_600_ was recorded every 10 min after a short vigorous shaking. OD_600_ obtained from wells containing the supernatant of Bt ΔpapR P*plcA*’-*lacZ* (a lecithinase minus strain) were substracted from the OD_600_ corresponding to the strains of interest. The resulting OD_600_ were then plotted as a function of time and the slope of each curve was determined from the linear part of the curve. A specific activity was determined using the following formula: slope/[OD_600_ of the culture at the time of sampling × volume of supernatant used in the assay (L)]. This formula was adapted from (Taniguchi et al., 2009). Each assay was repeated at least twice.

### Analysis of the Wild-Type and Δ*codY* Cell Culture Supernatants Using Mass-spectrometry

To facilitate detection of PapR in a peptide-poor medium, Bt 407^-^, Bt ΔcodY and Bt ΔpapR P*plcA*’-*lacZ* cells were grown at 37°C in S medium (1.7% synthetic broth AOAC, 0.2% NaCl) supplemented with 0.3% glucose instead of LB. In this medium, the expression profile of the PlcR-dependent gene *plcA* was similar to that in LB in the strains used (data not shown). 1 mL samples were harvested from cell cultures by centrifugation 30 min after entry into stationary phase. The supernatant was then filter-sterilized and kept at -20°C until analysis.

Peptides produced in S medium + glucose were isolated from 1 ml of supernatant by solid-phase extraction on StataX cartridges (Phenomenex) using a 50% acetonitrile solution for the elution step. Peptides were then concentrated by evaporation. Dried fractions were resuspended in 40 μl of 0.1% TFA, 2% acetonitrile and peptides abundance were measured by LC–MS/MS on an LTQ-Orbitrap mass spectrometer at the PAPPSO platform (http://pappso.inra.fr; See Supplementary Material and Methods for details).

### Analysis of the Opp Content in Membrane-Enriched Fractions of Wild-Type and Δ*codY* Cells Using Mass-spectrometry

#### Membrane-Enriched Fraction Preparation

Bt 407^-^ and Bt ΔcodY cells were grown at 37°C in LB and samples were collected by centrifugation 1 h before (t-1), at (t0), and 1 h after (t1) the transition from exponential to stationary phase (100, 50, and 50 mL, respectively). The pellets were washed once with PBS and stored at -20°C until further processing. The pellets were thawed, resuspended in lysis buffer [KPO_4_ pH6.6 100 mM, lysozyme 100 μg/mL, anti-protease cocktail (Roche) 1x] at an OD_600_ = 35 and incubated at 37°C for 30 min. The suspensions were sonicated on ice and sonication efficiency was verified by microscopic observation. A nuclease cocktail (DNAseI 10 μg/mL, RNaseA 10 μg/mL, MgCl_2_ 1 mM, CaCl_2_ 1 mM was added to the samples that were then incubated on ice for 1 h. The suspensions were centrifuged at 5000 *g* for 10 min at 4°C and the supernatant was then further centrifuged at 100000 *g* for 1 h at 4°C. The pellets were then resuspended in Laemmli sample buffer (Sigma) and kept at 4°C overnight until completely solubilized before protein concentration was measured using the 2D Quant kit (GE Healthcare). This experiment was repeated three times and 10 μg of proteins from each sample were then loaded on 12% SDS-polyacrylamide gels poured together form the same master mix. A short migration was then performed.

#### Protein In-gel Digestion

Each lane of short migration was cut and washed for 15 min with an acetonitrile/100 mM ammonium bicarbonate mixture (1:1). Digestion was performed in 50 mM ammonium bicarbonate pH 8.0 and the quantity of modified trypsin (Promega, sequencing grade) was 0.1 μg per sample. Digestion was achieved for 6 h at 37°C. The supernatant was conserved. Peptides were extracted by 5% formic acid in water/acetonitrile (v/v). Supernatant and extracted tryptic peptides were dried and resuspended in 50 μl of 0.1% (v/v) formic acid and 2% (v/v) acetonitrile. Proteins were detected and quantified by spectral counting approach by LC-MS/MS on a Q-Exactive mass spectrometer at the PAPPSO platform (http://pappso.inra.fr; See Supplementary Material and Methods for details). The raw data were submitted to the Pride database (Vizcaino et al., 2013) as a Proteom Xchange dataset (http://www.ebi.ac.uk/pride/archive/projects/PXD003311).

For ease of reading we removed “BTB_c” or “BTB_RS” from the locus tags and only kept the number associated.

The threshold for being referenced in Supplementary Table S5 was a mean number of spectra of at least 2 in at least 1 condition for the wild-type and the Δ*codY* samples.

### Peptide Synthesis

Synthetic PapR_7_ (ADLPFEF) and PapR_27_ (DTAFEKSQIISHNDQEVQVAADLPFEF) were ordered from Genscript at a purity >95% and were resuspended in water.

## Results

### PlcR-Dependent Genes are Down-regulated in a Bt Δ*codY* Mutant

We decided to construct a Δ*codY* mutant in strain Bt 407^-^ in order to examine the effect of this regulator in a genetic background in which the PlcR-PapR quorum-sensing system has been extensively characterized (for a review see Slamti et al., 2014). This strain is also capable of completing a full infectious cycle in our insect model *Galleria mellonella*. After unsuccessful attempts at disrupting *codY* by homologous recombination, it appeared that we could only delete *codY* in the presence of a copy of the gene in *trans*. We obtained the Bt ΔcodY strain after curing the cells of the plasmid carrying the ectopic copy of *codY* (see Materials and Methods). We cannot exclude that generation of this mutant might have resulted in the selection of one or more suppressor mutations elsewhere in the genome. Δ*codY* mutant cultures presented a slower growth rate in exponential phase and reached lower OD_600_ during stationary phase than wild-type cell cultures (Supplementary Figure S1). The mutant cells look also more elongated and thinner than wild-type cells and seem to present a chaining phenotype, in particular during exponential phase (Supplementary Figure S2). Disruption of the *codY* gene resulted in a drastic loss of hemolytic and lecithinase activities on sheep blood and egg yolk agar plates, respectively (Supplementary Figure S3). These activities are due to the production of PlcR-dependent gene products (Salamitou et al., 2000; Slamti et al., 2004). Introducing p1618K-P*xyl*’-*codY*, a plasmid carrying the *codY* gene under the control of the xylose-inducible promoter P*xyl*, in the Δ*codY* mutant restored the hemolytic and lecithinase activities of this strain (Supplementary Figure S3), confirming that the loss of these activities was due to the deletion of *codY*. These phenotypes are in agreement with what has been previously reported for *B. cereus* (Frenzel et al., 2012; Lindback et al., 2012).

**FIGURE 1:**
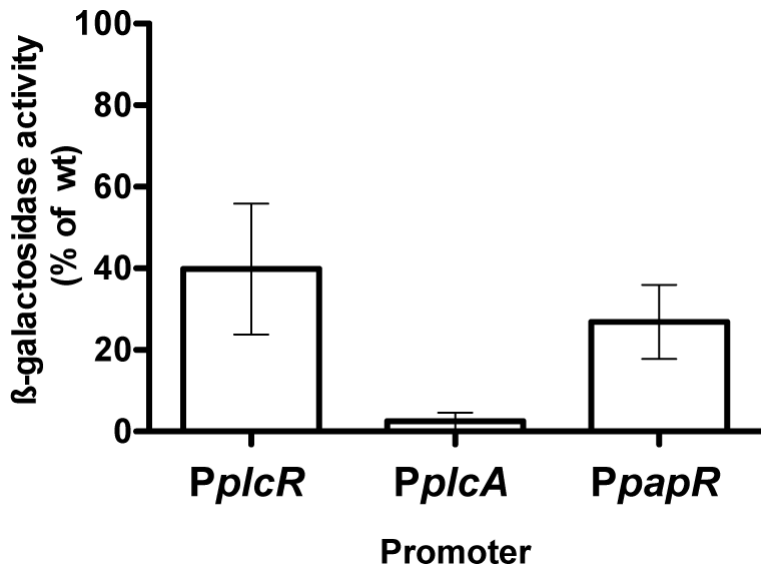
**Expression of PlcR-dependent genes in Bt ΔcodY compared to the wild-type strain.** Δ*codY* and wild-type cells harbor a transcriptional fusion between the promoter of interest and the *lacZ* gene. Cells were grown in LB at 37°C and harvested 2 h after entry into stationary phase. The β-galactosidase specific activity was then assayed. The results are the mean values of at least two independent experiments, and the error bars represent standard deviation.

We also measured the transcriptional activity of three PlcR-dependent promoters, fused to the reporter gene *lacZ*, in wild-type and Δ*codY* cells (**Figure 1**). The activity of the *plcR* and *papR* promoters is reduced by 60 and 70% in the Δ*codY* mutant compared to wild-type cells, respectively. In contrast, the activity of *plcA*, encoding the PI-phospholipase PlcA, is almost abolished in the mutant compared to the wild-type. Transcription of *plcA* was restored in Δ*codY* cells complemented with a copy of *codY* in *trans* (Supplementary Figure S4). In aggregate, these results show that CodY positively regulates the transcription of PlcR-dependent genes in Bt 407^-^, either indirectly or by direct interaction with the promoter of *plcR* or *papR*.

### CodY Controls the Activity of PlcR

**FIGURE 2:**
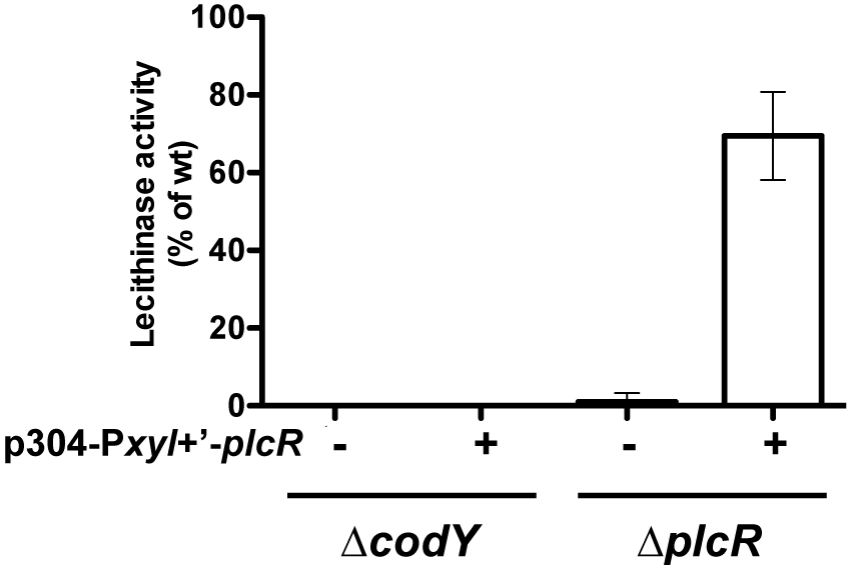
**Lecithinase production in Bt ΔcodY compared to the wild-type strain when *plcR* is overexpressed.** Δ*codY* and Δ*plcR* cells harbor (+) or not (-) the p304-P*xyl*+’-*plcR* vector carrying a transcriptional fusion between the xylose-inducible promoter P*xylA* and the *plcR* gene. Filter-sterilized supernatants from samples harvested 1 h after entry into stationary phase were assayed for lecithinase production. The results are the mean values of two independent experiments, and the error bars represent standard deviation.

As *plcR* is autoregulated, we needed to determine if CodY was involved in the transcriptional activation of *plcR* or in the activity of the protein itself. We constructed p304-P*xyl*+’-*plcR*, a plasmid carrying the *plcR* gene under the control of a modified version of the xylose-inducible promoter P*xyl* (see Materials and Methods). This allowed us to uncouple *plcR* transcription from the activity of its product. p304-P*xyl*+’-*plcR* was introduced in Bt ΔplcR and Bt ΔcodY. Lecithinase production was then assayed in these strains and compared to that of the wild-type strain (**Figure 2**). As *plcB*, the gene encoding the lecithinase PC-PLC, is under the control of PlcR and is absent in the supernatant of a Δ*plcR* mutant (Agaisse et al., 1999; Gohar et al., 2002, 2008), the activity of its product reflects the activity of the regulator. No lecithinase activity could be detected at t1 (1 h after the onset of stationary phase) in the Δ*codY* and Δ*plcR* mutants. Lecithinase activity was restored to almost wild-type levels in Bt ΔplcR (p304-P*xyl*+’-*plcR*) showing that our assay was functional, i.e., that *plcR* is transcribed and active in this strain. However, we could not detect any lecithinase activity in Bt ΔcodY (p304-P*xyl*+’-*plcR*) indicating that PlcR is not active in this strain and therefore that CodY controls PlcR activity.

### Activity of PlcR is Restored by Expressing the Mature form of PapR Inside Δ*codY* Cells

**FIGURE 3:**
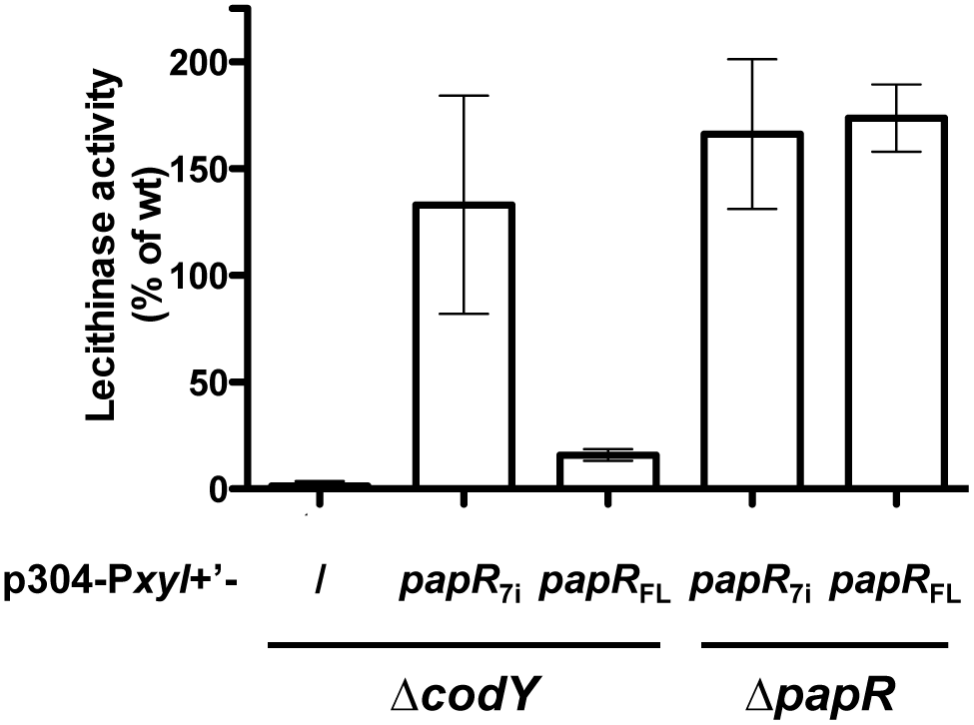
**Lecithinase production in Bt ΔcodY compared to the wild-type strain when the mature form of PapR is produced inside the cells.** Δ*codY* and Δ*papR* cells harbor the p304-P*xyl*+’ vector carrying a transcriptional fusion between the xylose-inducible promoter P*xylA* and either *papR_7i_*, coding for the mature form of PapR (7 aa), or *papR_FL_*, coding for full length PapR (including the signal sequence). / indicates that the cells are devoid of these plasmids. Filter-sterilized supernatants from samples harvested 1 h after entry into stationary phase were assayed for lecithinase production. The results are the mean values of two independent experiments, and the error bars represent standard deviation.

It has been shown that the activity of PlcR directly depends on the binding of the PapR peptide (Slamti and Lereclus, 2002). We investigated whether loss of the PlcR-dependent phenotypes in the *codY* mutant was due to an effect of CodY on either step of PapR production (export, maturation, or import). We have already shown that *papR* transcription was strongly reduced, but not abolished, in Δ*codY* cells (**Figure 1**). We introduced p304-P*xyl*+’-*papR*_7i_ or p304-P*xyl*+’-*papR*_FL_ in the Δ*codY* and Δ*papR* mutant cells. The former plasmid allows the production of the mature 7 aa form of PapR directly inside the cells. The latter encodes the full-length version of PapR, including the signal sequence. We then assayed the lecithinase activity of these strains and compared it to that of the wild-type strain (**Figure 3**). As above, no lecithinase activity could be detected at t1 in the Δ*codY* mutant. The lecithinase activity for Δ*papR* cells was very low and the measurements obtained for this mutant were used to normalize the results (see Materials and methods). Therefore the data corresponding to this strain could not be plotted on this graph. **Figure 3** shows that lecithinase activity was above wild-type levels in Bt ΔpapR P*plcA*’-*lacZ* (p304-P*xyl*+’-*papR*_7i_) and Bt ΔpapR P*plcA*’-*lacZ* (p304-P*xyl*+’- *papR*_FL_) showing that our assay was functional, i.e., that *papR*_7i_ and *papR*_FL_ are transcribed and active in these strains. The same result was observed for Bt ΔcodY (p304-P*xyl*+’-*papR*_7i_) indicating that expressing the mature form of PapR inside Δ*codY* cells is enough to restore the production of the PlcR-dependent protein PC-PLC. In contrast, lecithinase production was only 16% of that of the wild-type in Bt ΔcodY (p304-P*xyl*+’-*papR*_FL_) at t1. Since PapR_FL_ is not able to complement the *codY* mutation, these data suggest that export and/or maturation and/or import of PapR is affected in Δ*codY* mutant cells.

### PapR_7_ is Less Abundant in the Culture Supernatant of a Δ*codY* Mutant than in the Culture Supernatant of Wild-Type Cells

**FIGURE 4:**
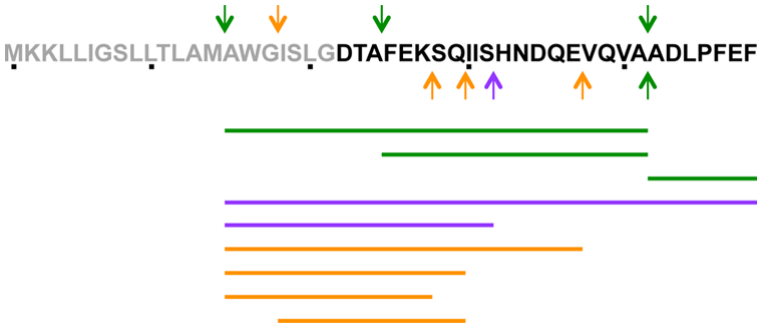
**Schematic representation of the presence of PapR-derived peptides in the supernatant of Bt ΔcodY compared to the wild-type strain.** The culture supernatant of Δ*codY* and wild-type cells was filter-sterilized before concentration and injection in an LTQ-Orbitrap for LCMS/MS analysis. Cells were grown in synthetic broth + glucose at 37°C. Samples were collected 0.5 h after entry into stationary phase. The arrows on the PapR peptide sequence indicate the main cleavage sites (in N-terminus, above the sequence and in C-terminus, below the sequence) deduced from the peptides found in the supernatant of both strains. The lines below the peptidic sequence schematize the peptides found in the supernatants. Green refers to cleavage sites and peptides common to Δ*codY* and wild-type, purple are specific to wild-type and orange to Δ*codY*.

To determine which step of PapR production was controlled by CodY, we examined if the signaling peptide could be detected in the supernatant of Bt ΔcodY. The supernatant of wild-type and Δ*codY* cells was harvested at t0.5 and filter-sterilized before being subjected to LC-MS/MS analysis. The arrows on the PapR peptide sequence in **Figure 4** indicate the main cleavage sites deduced from the peptides found in the supernatant of both strains. **Table 3** and **Figure 4** show the sequence and abundance of each peptide found either in the wild-type or the Δ*codY* culture supernatant or in both. The most commonly found N-terminal end is the alanine in position 15 in both cases. However, the similitude does not much extend further as the only other common cleavage site is after the alanine in position 41 (green arrows in **Figure 4**). The PapR-derived peptidic sequences found in the two supernatants are otherwise different, suggesting a different processing between the strains (**Table 3** and compare the purple and orange arrows in **Figure 4**). It is also important to note that the abundance of PapR_7_ (ADLPFEF) was lower in the supernatant of the Δ*codY* cells than in that of the wild-type strain, as this could result in the inability to reach the concentration required for the activation of PlcR.

**Table 3:** PapR-derived peptides detected in wild-type and Δ*codY* cell culture supernatants.

Sequence	Best Evalue^a^	m/z (th)^b^	Relative Δ*codY* area (% of wt)	Wild-type area^c^	Δ*codY* area^c^
**Peptides found in both supernatants**					
AWGISLGDTAFEKSQIISHNDQEVQVA	5.80E-03	981.821(3)	9,4	3.22E+08	3.03E+07
FEKSQIISHNDQEVQVA	1.60E-06	986.492(2)	5,6	2.89E+07	1.61E+06
ADLPFEF	>0.05	838.399(1)	11.9	2.43E+07	2.90E+06
**Peptides found exclusively in the wild-type cell culture supernatant**					
AWGISLGDTAFEKSQIISHNDQEVQVAADLPFEF	1.50E-10	1254.948(3)	/	9.24E+06	ND
AWGISLGDTAFEKSQIIS	3.10E-04	961.996(2)	/	2.89E+07	ND
**Peptides found exclusively in the Δ*codY* cell culture supernatant**					
AWGISLGDTAFEKSQIISHNDQE	6.60E-05	849.410(2)	/	ND	1.28E+07
AWGISLGDTAFEKSQ	3.80E-13	805.397(2)	/	ND	5.52E+07
AWGISLGDTAFEK	6.60E-13	697.581(2)	/	ND	4.69E+07
ISLGDTAFEKSQ	7.10E-07	648.328(2)	/	ND	4.42E+07

*^a^Best peptide Evalue calculated by the X!tandem software.*

*^b^Ion masses selected for fragmentation (charge state).*

*^c^Manually Extracted Ions Current using Qualbrowser tools.*

*ND, Not detected.*

### The Culture Supernatant of the Bt ΔcodY Strain Contains Enough PapR to Complement Δ*papR* Cells

**FIGURE 5:**
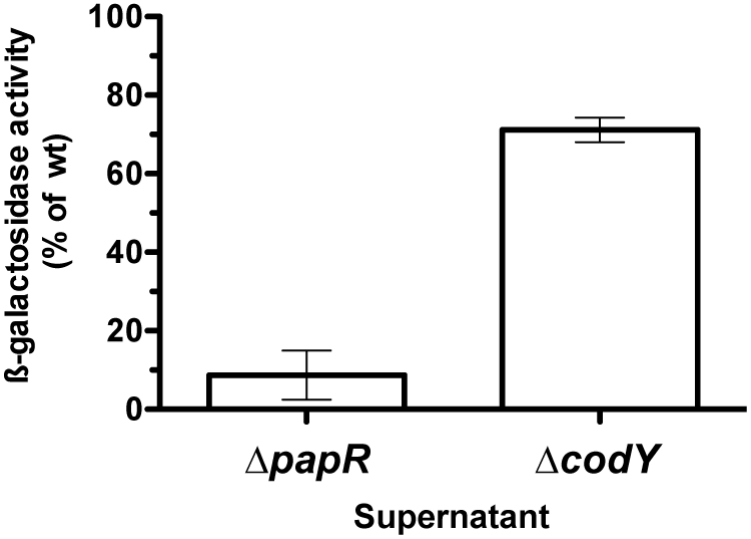
**Extracellular complementation assay of Δ*papR* cells with conditioned medium from Δ*codY* compared to wild-type cells.** The culture supernatant of Δ*codY*, Δ*papR* and wild-type cells was filter-sterilized before being used to resuspend Δ*papR* cell pellets. The latter harbor a chromosomal transcriptional fusion between the promoter of *plcA* and *lacZ*. Cells were grown in LB at 37°C. All samples were collected 0.5 h after entry into stationary phase. β-galactosidase production was assayed after resuming growth for 1.5 h. The results are the mean values of two independent experiments, and the error bars represent standard deviation.

We then examined if the amount of PapR present in the Δ*codY* culture supernatant was able to complement a Δ*papR* mutant for the expression of a PlcR-dependent gene. β-galactosidase production was assayed in Bt ΔpapR P*plcA*’-*lacZ* cells grown in the presence of supernatant harvested from wild-type, Δ*codY* or Δ*papR* cell cultures at t0.5. **Figure 5** shows that the activity of the *plcA* promoter in Bt ΔpapR P*plcA*’-*lacZ* cells grown in the presence of supernatant from Δ*papR* cells is about 10% of its activity compared to when they are grown in conditioned medium from wild-type cells. This is consistent with what has been previously reported (Slamti and Lereclus, 2002). In contrast, supernatant from Δ*codY* cell cultures was able to partially complement Bt ΔpapR P*plcA*’-*lacZ* cells and to induce transcription at the *plcA* promoter to 70% of its activity compared to when cells are cultured in supernatant from wild-type cells. This indicates that Bt ΔcodY cells secrete enough PapR to activate the PlcR regulator.

### Import of PapR is Impaired in Δ*codY* Cells

**FIGURE 6:**
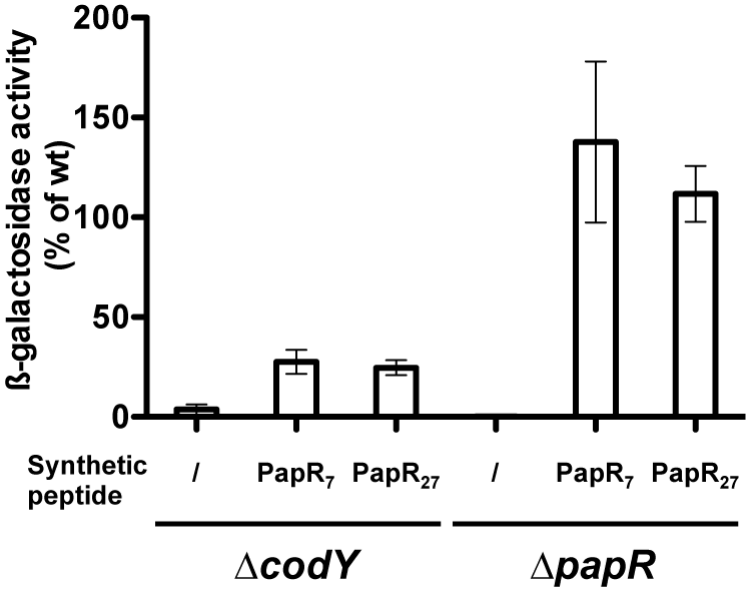
**Extracellular complementation assay of Δ*codY* cells with synthetic PapR peptides.** Δ*codY* and Δ*papR* cell cultures were split at the entry of stationary phase and synthetic peptides were added when indicated at a final concentration of 5 μM. The cells harbor the pP*plcA*’-*Z* plasmid, carrying a transcriptional fusion between the promoter of *plcA* and *lacZ.* Cells were grown in LB at 37°C. Samples were collected for β-galactosidase assay 2.5 h after addition of the peptides. The sequence of the PapR peptides is indicated in Section “Materials and Methods.” The results are the mean values of two independent experiments, and the error bars represent standard deviation.

To directly assess if PapR could be reimported in the Δ*codY* mutant, we measured β-galactosidase production in Bt ΔcodY (pP*plcA*’-*Z*) cells grown in the presence of synthetic PapR_7_ peptide (**Figure 6**). The results show that the ß-galactosidase activity in these cells is 27% of that of Bt (pP*plcA*’-*Z*), whereas it reaches 137% in Bt ΔpapR (pP*plcA*’-*Z*) cells cultured in the same conditions. This indicates that deletion of *codY* impairs the import of the mature form of PapR in Bt cells.

Furthermore, synthetic PapR_27_, which corresponds to full length PapR (excluding the predicted signal sequence) also induced the same activation of P*plcA*’-*Z* in Δ*codY* as did PapR_7_, suggesting that maturation of PapR can occur in this strain. PapR_7_ and PapR_27_ also induced the same activation of P*plcA*’-*Z* in Δ*papR* cells.

### OppA is Involved in -but is Dispensable for- the Import of PapR

**FIGURE 7:**
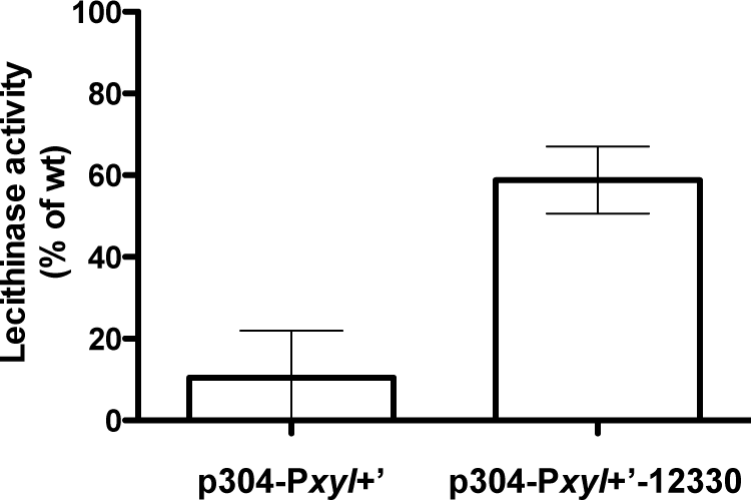
**Lecithinase activity of Δ*codY* cells overexpressing *oppA* in the presence of synthetic PapR peptide.** Δ*codY* cells harbor the empty p304-P*xyl*+’ vector or express the *oppA* gene under the control of the P*xyl+* promoter (p304-P*xyl*+’-12330). The cultures were split 0.5 h before the transition into stationary phase (t-0.5) and the synthetic peptide PapR7 was added at a final concentration of 5 μM. Cells were grown in LB supplemented with xylose at 37°C. Samples were collected for lecithinase assay 2.5 h after addition of the peptide. The sequence of the peptide is indicated in Section “Materials and Methods.” The results are the mean values of two independent experiments, and the error bars represent standard deviation.

The pore components required for proper activation of the expression of the PlcR regulon were shown to be OppB-C-D-F (corresponding to proteins 12340, 12350, 12360, and 12370, respectively; Gominet et al., 2001). However, no information was reported about the SBP. We examined if OppA (protein 12330) was responsible for the import of PapR. We assayed lecithinase production of Δ*codY* cells in which we overexpressed *oppA*. We showed that expression of *oppA* induces an increase in lecithinase activity in the Δ*codY* mutant in the presence of PapR7, compared to that of the strain in which the gene is not expressed (about 60 and 10% of the activity of wild-type samples, respectively; **Figure 7**). This strongly supports the direct involvement of OppA in the import of the peptide. We then deleted the *oppA* gene and assessed the hemolytic activity of the Bt ΔoppA strain on sheep blood agar plates as a reporter of PlcR activity. Contrary to what was reported previously for a Δ*oppB* mutant (Gominet et al., 2001), there was no obvious difference between the Δ*oppA* mutant and the wt strain (Supplementary Figure S5), indicating that, although OppA is involved in the import of PapR, other SBPs are able to fulfill the same function.

### Several Opp-Like Proteins are Differentially Detected in Δ*codY* Membrane-Enriched Fractions Compared to Wild-Type Fractions

**FIGURE 8:**
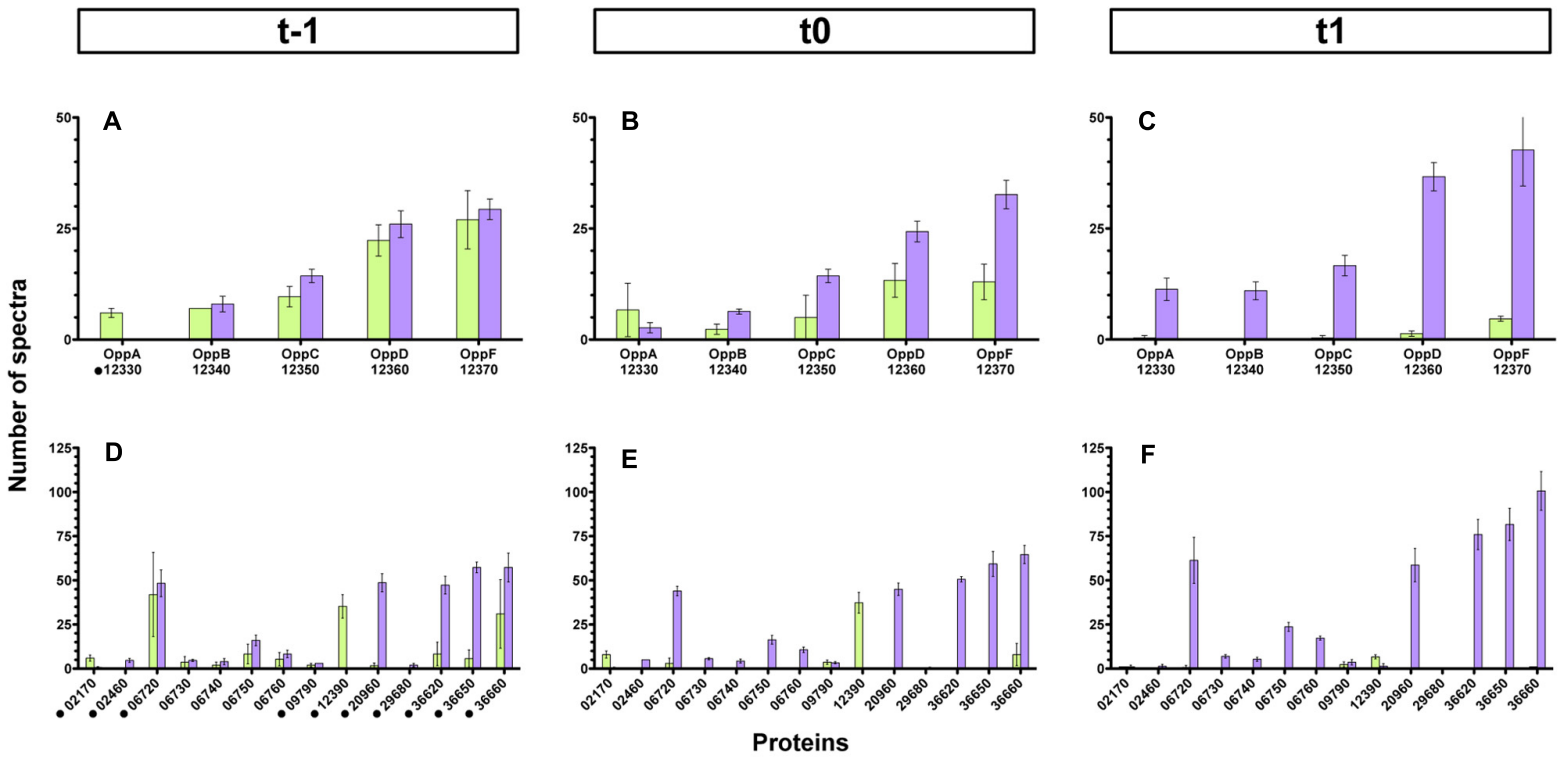
**Detection of Opp-like proteins in membrane-enriched fractions from Δ*codY* and wild-type cells.** Membrane-enriched fractions obtained from Δ*codY* and wild-type cells harvested 1 h before (t-1, **A,D**), at (t0, **B,E**), or 1 h after (t1, **C,F**) the transition into stationary phase were analyzed using mass spectrometry. The histograms represent the number of spectra detected in the samples for each protein indicated on the x-axis. The upper panels report the data for the components of the oligopeptide permease Opp of which the pore is required for PapR import. The lower panels report the data for the other Opp-like proteins detected. The results are the mean values of three independent experiments, and the error bars represent standard deviations. The black dots in **(A,D)** indicate an OppA-like protein. Purple bars, wild-type samples; green bars, Δ*codY* samples.

In order to assess which permease component involved in the import of PapR was affected by the *codY* mutation, and because Bt harbors 40 genes that are predicted to encode a component of potential oligopeptide permease systems (Supplementary Table S4), we chose to use a general approach. Membrane enriched fractions of wild-type and ΔcodY cells harvested 1 h before (t-1), at (t0), and 1 h after (t1) the transition from exponential to stationary phase were prepared and subjected to mass spectrometry analysis. Label free quantification of proteins was achieved using spectral counting which allows the relative quantification of proteins based on the number of spectra obtained with tryptic peptides in mass spectrometry. This quantification relies on the fact that the more of a particular protein is present in a sample, the more MS spectra are detected for peptides from that protein. We identified a total of 1366 proteins in all the samples (the complete results are available on the PRIDE database (Vizcaino et al., 2013) as a Proteom Xchange dataset under the submission number PXD003311). 22% of these proteins were predicted to be membrane-associated using the PSORTb v3.0 software (Yu et al., 2010). Nineteen Opp-like proteins passed our detection threshold. They are listed in Supplementary Table S5. We could identify all the components of the permease required for the import of PapR (i.e., OppB-C-D-F). The results show that there was no difference in the detection of the pore components at t-1 in wt and Δ*codY* cells (**Figure 8A** and Supplementary Table S5). However, there was less OppB-C-D-F in the *codY* mutant than in the wt strain at t0, and, at t1, almost none of these proteins were detected in the Δ*codY* membrane-enriched fraction (**Figures 8B,C** and Supplementary Table S5). In contrast, OppA (protein 12330) seems to follow a different pattern. **Figure 8A** shows that more OppA was detected in the Δ*codY* sample than in the wt preparation. At t0 there was no significant difference between the samples, and, at t1, no OppA was detected in Δ*codY* membrane-enriched preparation (**Figures 8B,C** and Supplementary Table S5).

Interestingly, other Opp-like proteins were differentially detected in the wild-type and Δ*codY* samples. Panels D, E and F of **Figure 8** (as well as Supplementary Table S5) recapitulate all these proteins. Protein 12390 was significantly more represented in the Δ*codY* samples than in the wt preparations at all times. However, its number of spectra was strongly reduced between t0 and t1. To a lesser extent, protein 02170 was also significantly more represented in the Δ*codY* samples than in the wt preparations, except at t1 where it was no longer detected. In contrast, proteins 20960, 36620, 36650, and 36660 saw their level drastically reduced in the Δ*codY* membrane-enriched fraction compared to their wt level, at all times. The level of protein 06720 was also drastically reduced in the Δ*codY* background compared to its wt level, but only at t0 and t1. Proteins 06730, 06740, 06750, and 06760 follow the same pattern but with less amplitude. The other proteins show little -or no significant- difference between the samples.

### Ectopic Over-expression of Selected OppA-Like-Encoding Genes Partially Restores the Hemolytic Phenotype of Δ*codY* Cells

**FIGURE 9:**
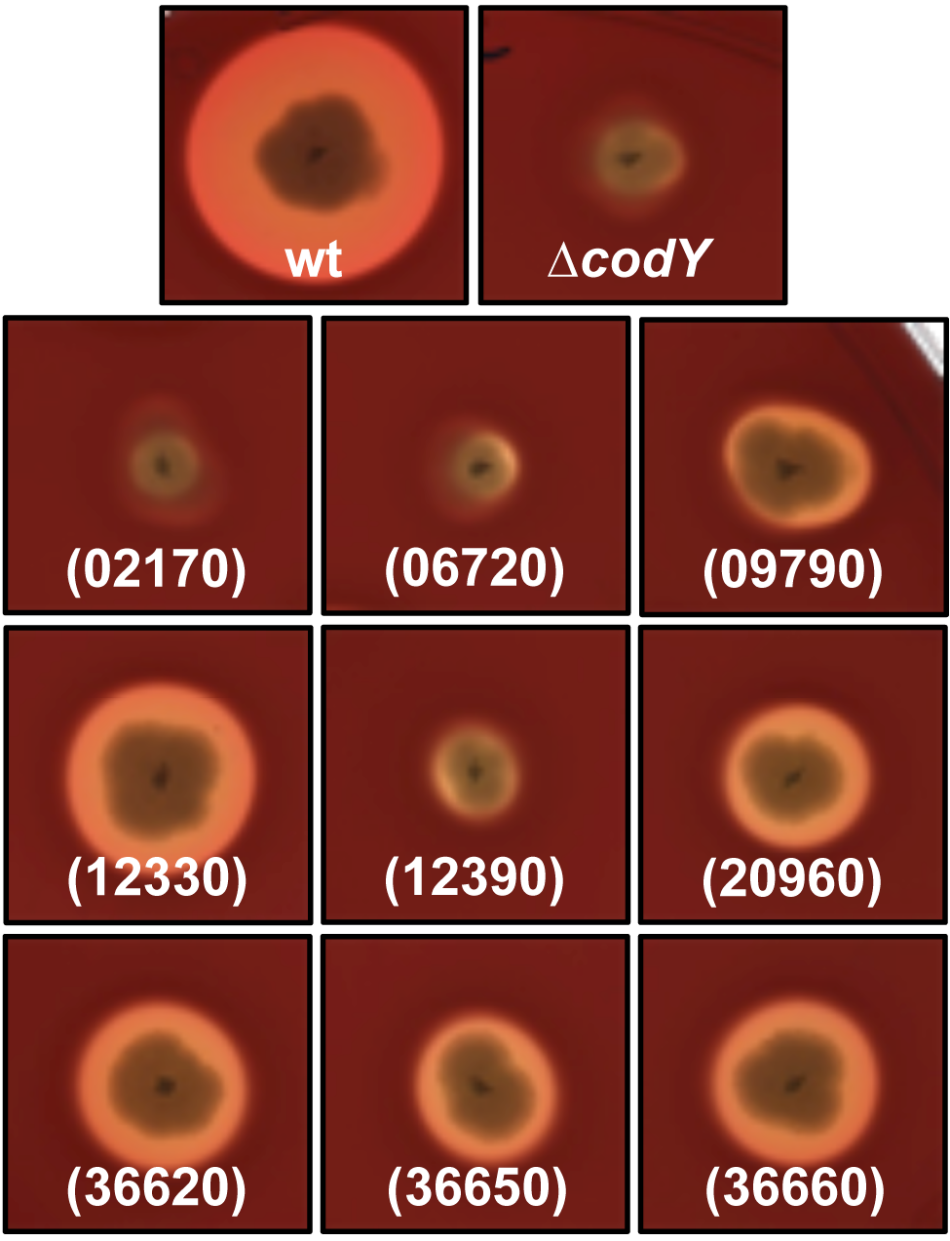
**Hemolytic activity of Δ*codY* cells over-expressing *oppA* and *oppA*-like-encoding genes.** Cells were patched onto a Columbia sheep blood agar plate supplemented with xylose and grown at 37°C for 16 h. The cells either harbor the empty vector p304-P*xyl+* or overexpress our gene of interest under the control of a xylose-inducible promoter. wt, Bt 407- (p304-P*xyl+*); Δ*codY*, Bt ΔcodY (p304-P*xyl+*); (02170), Bt ΔcodY (p304-P*xyl+*-02170); (06720), Bt ΔcodY (p304-P*xyl+*-06720); (09790), Bt ΔcodY (p304-P*xyl+*-09790); (12330), Bt ΔcodY (p304-P*xyl+*-12330); (12390), Bt ΔcodY (p304-P*xyl+*-12390); (20960), Bt ΔcodY (p304-P*xyl+*-20960); (36620), Bt ΔcodY (p304-P*xyl+*-36620); (36650), Bt ΔcodY (p304-P*xyl+*-36650); (36660), Bt ΔcodY (p304-P*xyl+*-36660). This picture is representative of at least three independent experiments.

In order to assess if we could restore the import of PapR in Δ*codY* cells, we over expressed seven genes encoding for OppA-like proteins that were less abundant in Bt ΔcodY than in the wt strain [06720, 12330 (OppA), 20960, 36620, 36650, 36660], according to the results presented above. We also overexpressed two genes encoding OppA-like proteins that were more abundant in Bt ΔcodY than in the wt strain (02170 and 12390) as a control. We then assayed the hemolytic activity of these strains. The results presented in **Figure 9** show that over-expression of some OppA-like-encoding genes, but not all, partially restored the hemolytic activity of Δ*codY* cells. We observed the same results when monitoring the lecithinase activity of these cells (Supplementary Figure S6). Over-expression of the 02170 and 12390 genes did not induce hemolysis of the red blood cells. This is in agreement with the fact that their corresponding proteins were more abundant in ΔcodY than in wt preparations (**Figure 8**, lower panels). Interestingly, over-expression of gene 06720 does not have any effect on the hemolytic phenotype of the Δ*codY* cells, despite the fact that it was more abundant in the wild-type than in the Δ*codY* samples (**Figure 8**, lower panels). This result suggests that this protein is probably not involved in the uptake of PapR. In contrast, over-expression of gene 12330, which encodes OppA, and of genes 20960, 36620, 36650 and 36660, which are more abundant in the wt samples than in the Δ*codY* samples (**Figure 8**, lower panels), induce an increase in the hemolytic activity of the Δ*codY* cells, suggesting that they are all able to bind PapR and allow its import.

## Discussion

We assessed the mechanism underlying the control of the global regulator CodY on the expression of the PlcR-dependent virulence genes in Bt, an entomopathogen of the Bc group. Involvement of CodY in the expression of these genes had been demonstrated in two Bc strains, but no mechanistic explanation has been reported (Frenzel et al., 2012; Lindback et al., 2012). Several unsuccessful attempts at deleting the *codY* gene in Bt 407^-^, our reference strain, had been made in our laboratory and the difficulties we encountered to generate our mutant strongly suggest that this gene is essential for the survival of this strain in our laboratory conditions. A *codY* deletion mutant in Bt 407^-^ could only be generated in a strain harboring an ectopic copy of the gene. The loss of the plasmid carrying this copy resulted in obtaining a marker-less Δ*codY* mutant. It is possible that the mutant we obtained contains mutations allowing for the growth of Bt in the absence of *codY*. It has been reported that CodY was essential in two *Streptococcus pneumoniae* strains (Caymaris et al., 2010). The authors speculated that *codY* inactivation might lead to an increase in iron uptake and that iron toxicity might explain the lethality of this mutation. To survive in the presence of the *codY* knock-out, the cells contained additional mutations in the putative iron transporters. In Bt, we showed that the hemolytic and lecithinase activities linked to PlcR-regulated genes were abolished in the Δ*codY* mutant and were restored when the *codY* mutation was complemented *in trans*. This indicates that the regulatory link between PlcR and CodY was not affected by these hypothetical suppressor mutations. There is no mention of the putative essential nature of the *codY* gene in the previous reports using Δ*codY* mutants in 3 Bc strains of differing origins (Hsueh et al., 2008; Frenzel et al., 2012; Lindback et al., 2012). This might reflect a different requirement for CodY in these genetic backgrounds.

In agreement with results reported in Bc (Frenzel et al., 2012; Lindback et al., 2012), we showed that three PlcR-dependent promoters were downregulated in Bt ΔcodY compared to the wild-type strain. Our study shows that, although the *papR* gene is less transcribed in Bt ΔcodY than in its wild-type parent, PapR-derived peptides were found in the supernatant of the mutant cells. However, in addition to the lower concentration in PapR derivatives, the sequences of the peptides detected were different between the mutant and the wild-type strain. It appeared that PapR was cleaved in Bt ΔcodY at sites that were not used in the wild-type strain. It has been reported that the PlcR-controlled NprB peptidase was, at least in part, responsible for the maturation of PapR (Pomerantsev et al., 2009). However, PapR_27_ was able to activate PlcR in Δ*papR* cells, suggesting that other peptidases might be involved in the maturation process, at least before the positive autoregulatory loop is triggered and NprB is produced. *nprB* is among the most downregulated genes in the Bc Δ*codY* mutant compared to the wild-type cells in the transcriptomic analysis reported by Lindback et al. (2012). It is then unlikely that it could fulfill its function in Δ*codY* cells in a similar manner as in wild-type cells. We hypothesize that, in Δ*codY* cells, NprB is rare and that this could facilitate the access of other peptidases to PapR, including peptidases that would usually be less abundant. We also noted that there was no concordance between the predicted cleavage site after the putative signal sequence and the peptides found in the supernatant of both strains. We did not detect peptides with D22 in the N-terminal position, as expected from the prediction. Aside from the ADLPFEF peptide, we only found peptides with A15 or F25 at their N-terminal end in the wild-type cell culture supernatant. This suggests that the signal peptide might have been improperly predicted and might be defined as the first 14 amino acids of the PapR sequence.

Expressing the mature form of PapR inside Δ*codY* cells was enough to restore the production of PlcR-dependent proteins. We also showed that the concentration of PapR in a Δ*codY* cell culture supernatant is sufficient to activate PlcR in Δ*papR* cells. These results indicate an import deficiency in Bt ΔcodY compared to wild-type. Import of PapR has been previously investigated and it is known that it requires the pore components of the Opp permease, OppB-C-D-F (Gominet et al., 2001). However, no data was reported concerning the involvement of the OppA SBP. We showed that an *oppA* deletion mutant has a hemolytic phenotype similar to that of its wild-type parent on sheep blood agar plates, indicating that, even though we demonstrated that OppA was involved in the import of PapR, the cell produces other SBP with the same function. Bt carries 40 genes that encode proteins potentially involved in peptide import. Five *loci* encoding all the components of oligopeptide permeases were identified, including the one encoding OppA-B-C-D-F mentioned above. Aside from the latter, no function has been described for the other putative transporters. In addition to transporting peptides, members of the Opp family are also able to import nickel. Without functional assays, it is difficult to assign them a role. Interestingly, eight OppA-like proteins seem orphan, which is not common among bacteria. In order to understand the effect of the *codY* mutation on the production of the Opp permease and the other Opp-like proteins we undertook a proteomics approach and compared the protein contents of membrane-enriched fractions of Δ*codY* cells to that of wild-type cells. Among the 1366 proteins detected in all the samples 19 are putative components of oligopeptide permeases. It was previously shown that CodY impacts the expression of genes encoding oligopeptide permeases in *Lactococcus lactis* (Guédon et al., 2001) and *B. subtilis* (Slack et al., 1995; Molle et al., 2003). In a Bc Δ*codY* strain however, several Opp-like-encoding genes were downregulated whereas others were upregulated (Lindback et al., 2012).

Mass spectrometry analysis of membrane-enriched fractions allowed us to detect 19 out of the 40 Opp-like proteins predicted to be encoded in the genome of Bt. Some of these proteins were either upregulated in Δ*codY* cells compared to wild-type or were not affected by the mutation. Ten of these proteins were downregulated in Bt ΔcodY compared to the wild-type strain, including the components of the Opp permease, except OppA in late exponential phase. Results of the overexpression of the genes encoding the OppA-like proteins with this profile in the Bt ΔcodY background showed that some proteins [09790, 12330 (OppA), 20960, 36620, 36650, and 36660] were able to partially restore PlcR-dependent phenotypes in the mutant. It is worth noting that several OppA-like proteins are potentially able to bind PapR and to interact with its dedicated permease in order to allow its import in the cell. Proteins 02170 and 12390, that were less detected in Δ*codY* than in wt samples did not have any influence on PlcR activity, indicating that hemolysis restoration was not an artifact linked to the overexpression of any OppA-encoding gene. Remarkably, overexpression of gene 06720, whose abundance was higher in Δ*codY* than in wt preparations, did not affect the hemolytic activity of Δ*codY* cells.

Interestingly, and although the *opp* locus was reported as being transcribed as an operon (Gominet et al., 2001), there is a difference in the detection profile of OppA and the other components of the Opp permease. We are not able to explain this observation. We can, however, hypothesize that OppA is not present during exponential phase in wild-type cells to avoid the untimely entry of other peptides, such as Phrs, involved, for example, in triggering sporulation, and that the other OppA-like proteins that allow the import of PapR, might have a better affinity for the latter than for the Phrs. This would be in agreement with the need of functional SBPs during late vegetative growth to import and accumulate PapR in the bacterial cytoplasm. OppA is eventually produced and is able to import PapR, in order to reinforce the feedback loop.

In our study, we show that the positive effect that CodY exerts on the expression of PlcR-regulated genes depends on its effect on OppA-like proteins. However, we do not know if this effect is direct or not. It was recently shown that CodY indirectly activated the expression of *dtpT* (a putative oligopeptide permease in *B. subtilis*) *via* the direct repression of the gene encoding the repressor ScoC (Belitsky et al., 2015), also present in Bt. We cannot exclude that CodY influences the expression of *opp* genes indirectly in the same manner, or by activating another activator.

Involvement of CodY in the control of the import of PapR allows the PlcR-PapR QS system to integrate two types of external signals: the composition of the medium and the accumulation of the peptide. This mechanism adds another layer of complexity to the regulatory circuits governing QS in Bt. Indeed, it has been shown that CodY directly represses the transcription of *nprR*, a quorum sensor responsible for the ability of Bt to survive on insect cadavers after the bacteria have killed their host (Dubois et al., 2013). *nprR* is also under the positive control of PlcR. During stationary phase, Spo0A, the major regulator of sporulation, represses *plcR* transcription (Lereclus et al., 2000; Gominet et al., 2001). Our laboratory has shown that, *in vivo*, in the insect model of infection *Galleria mellonella*, a PlcR-dependent gene was activated during the virulent stage of the bacterium, then its expression decreased as the expression of an NprR-dependent gene increased, after the insect death (Dubois et al., 2012). The influence of CodY on the virulence of pathogens has been previously reported, as mentioned in the introduction, linking the virulent behavior of the bacterium to its metabolic state. Altogether, our data are in line with these reports and describe one of the mechanisms underlying these finely tuned regulatory circuits that control interconnected physiological stages to coordinate the behavior of the bacteria throughout growth.

## Author Contributions

Conceived and designed the study: LS, DL; designed the experiments: LS, DL; performed the experiments: LS, CL, CH, AG; constructed a mutant strain: EH; analyzed the data: LS, DL, CH, AG; wrote the paper: LS.

## Conflict of Interest Statement

The authors declare that the research was conducted in the absence of any commercial or financial relationships that could be construed as a potential conflict of interest.
