# Supplementary material for: CodY Regulates the Activity of the Virulence Quorum Sensor PlcR by Controlling the Import of the Signaling Peptide PapR in Bacillus thuringiensis
Source: Front Microbiol. 2016 Jan 6;6:1501. doi: 10.3389/fmicb.2015.01501 (PMC4701985; doi:10.3389/fmicb.2015.01501)
Supplement: Supplementary file 1 [file Data_Sheet_1.PDF]

CodY controls the activity of the virulence regulator PlcR by allowing the import of the signaling peptide PapR in *Bacillus thuringiensis*

Leyla Slamti[1]*, Christelle Lemy[1], Céline Henry[1,2], Alain Guillot[1,2], Eugénie Huillet[1] and Didier Lereclus[1]

[1]INRA, UMR1319 Micalis, Jouy-en-Josas, France and AgroParisTech, UMR Micalis, Jouy en Josas, France.
[2]INRA, UMR1319 PAPPSO, Jouy-en-Josas, France

***Corresponding author:**
Leyla Slamti
leyla.slamti@jouy.inra.fr
+33 1 34 65 24 83

SUPPLEMENTARY MATERIALS AND METHODS

DNA manipulation

Plasmid DNA was extracted from *E. coli* by a standard alkaline lysis procedure, using Promega or Qiagen kits. DNA fragments were purified using Promega or Qiagen kits. Chromosomal DNA was extracted from *B. thuringiensis* cells, harvested in mid-exponential growth phase, using the Qiagen Puregen Yeast/Bacteria kit. Restriction enzymes and T4 DNA ligase were purchased from New England Biolabs and used as recommended by the manufacturer. The oligonucleotide primers (Supplementary Table 1) used for PCR amplification were synthesized by Sigma-Aldrich. PCR was performed with a 2720 thermal cycler (Applied Biosystems). All constructs were systematically verified by PCR followed by sequencing of the region of interest. Nucleotide sequences were determined by Beckman Coulter Genomics.

Plasmid loss assay

Bt Δ*codY* (p1618K-P*xyl*'-*codY*) and Bt (p1618K-P*xyl*'-*codY*) cells were grown in LB at 37°C until the beginning of stationary phase, diluted 100x and grown in the same conditions again. Cells were then diluted 1000x and grown overnight at 30°C. Two growth cycles were performed again at 37°C. Serial dilutions were then plated on LB agar then patched on LB agar plates supplemented or not with kanamycin and incubated at 37°C. Loss of plasmid and the *codY* deletion were verified for Kan^S clones by PCR using primer pairs PR/xylout and codY7/codYDnBam, respectively.

PapR peptide relative quantitation on LTQ orbitrap

1 µl were loaded on a RSLC Pepmap100 C18 column (length 250 mm, 75 µm ID, 2 µm, 100 Å; Dionex). Peptide separation was performed at 300 nl/min over 43 min with a linear gradient of acetonitrile between 2 to 80 % in formic acid (0.1%). Eluted peptides were analyzed on-line on a LTQ-Orbitrap Discovery mass spectrometer (Thermo Fischer, San Jose). Ionization was performed on liquid junction with a spray voltage of 1.3 kV applied to a non-coated capillary probe (PicoTip EMITER 10 µm tip ID; New Objective, USA). Peptide ions were automatically analyzed by the data dependent method as follows: a) full MS scan (m/z 350–1500) on Orbitrap mass analyser, b) MS/MS on the most abundant precursors (mono, doubly and triply charged ions) on the linear ion trap with an exclusion window of 30 sec with classical peptide fragmentation parameters (Qz of 0.22, activation time of 50 ms, collision energy of 35%) and c) targeted CRM event on the linear ion trap by following the transition on m/z 838.4 to 539.3 corresponding to y4 ion produced by the ADLPFEF sequence with a normalized collision energy of 40%.
Native peptide were identified with X!TandemPipeline version 3.3.0 using the Uniprot protein sequences database of *Bacillus thuringiensis BT407*, associated to a proteomic contaminant database. The X!tandem search parameters included no cleavage specificity, variable oxidation of methionine, mass tolerance of 10 ppm for the parent ion M+H mass, and of 0.4 Da for the ion fragment. Identification results were filtered using E-value thresholds of 0.05 and 0.0025 for peptides and proteins, respectively, and a minimum of 2 peptides per protein.
Ratio of abundance corresponding to native PapR peptide between the wild-type and the Δ*codY* mutant were calculated using an ion current extraction in the full MS scan event for each validated peptide sequence by Xtandem! with Qualbrowser tools included in Xcalibur software (version 2.2.44, 11 sept 2011).

Protein Quantitation by spectral counting approach

Nano Liquid Chromatography (LC) Coupled to Mass Spectrometry (MS) in Tandem — A Q Exactive (Thermo Fisher Scientific) coupled to Eksigent 2Dnano LC (AB-Sciex, Massachusetts, USA) were used for the nano-LC-MS/MS analysis. 4 µl of sample were injected on the nanoLc-Ultra system (Eksigent) chain. Sample was loaded at 7.5 µl / min on the precolumn cartridge (C18, 5 µm, 120 Å, 20 mm Nanoseparations) and desalted with 0.1% formic acid. Then, peptides were separated with a gradient of acetonitrile on the reverse phase column C18 (stationary phase: C18 Biosphere, 3 µm; column: 75 µm i.d., 300 mm; Nanoseparations). Buffers were 0.1% formic acid in water (A) and 0.1% formic acid in acetonitrile (B). The peptide separation was achieved with a linear gradient from 5 to 35% B for 86 min at 300 nL/min^{-1} (Total gradient of 97 minutes). Eluted peptides were analyzed on-line with a Q Exactive mass spectrometer (Thermo Electron) using a nanoelectrospray interface. Ionization (1.8 kV ionization potential) was performed with stainless steel emitters (30 µm i.d.; Thermo Electron). Peptide ions were analyzed using Xcalibur 3.0.63 (Tune version 2.3) with the following data-dependent acquisition steps: (1) full MS scan (mass-to-charge ratio (m/z) 400 to 1400) and (2) MS/MS. Step 2 was repeated for the 8 major ions detected in step 1. Dynamic exclusion was set to 40 s. Lock mass option was chosen "best", MS resolution 70000 at m/z 400, auto gain control was 3e6, maximum injection time 250 ms. For MS2 the resolution was 17500 at m/z 400, auto gain control was 5e4 with maximum injection time of 120 ms, isolation window m/z = 3, normalized collision energy: 27, underfill ratio 0.5 %, intensity threshold 8.3e3. Charge state: 2, 3.

Data Processing and Bioinformatics Analysis — The Bt407 database was downloaded from Uniprot KB database site (www.uniprot.org, december 2014, 6858 protein entries). This database was merged and in conjunction with reverse and contaminant databases, were searched by X!Tandem (Sledge Hammer version 2013.09.01.1, http://www.thegpm.org/tandem/) using X!TandemPipeline (version 3.3.4) developed by PAPPSO platform (http://pappso.inra.fr/bioinfo/). Enzymatic cleavage was declared as a trypsin digestion with one possible miss-cleavage. Cys carboxyamidomethylation and Met oxidation were set to static and possible modifications, respectively. Precursor mass was 10 ppm and fragment mass tolerance was 0.02 Da. A refinement search was added with similar parameters except that semi-trypsic peptide and possible N-ter proteins. For data of proteomic, only peptides with an E value smaller than 0.1 were reported. Identified proteins were filtered and grouped using X!TandemPipeline (http://pappso.inra.fr/bioinfo/xtandempipeline/) according to: (1) A minimum of two different peptides was required with an E value smaller than 0.05, (2) a protein log (E value) (calculated as the product of unique peptide E values) smaller than 2.10− 3. These criteria led to a False Discovery Rate (FDR) of 0.05 % for peptide and protein identification. To take redundancy into account, proteins with at least one peptide in common were grouped. This allowed to group proteins of similar function. Within each group, proteins with at least one specific peptide relatively to other members of the group were reported as sub-groups (Washburn *et al.*, 2001). Label free quantification of proteins was achieved in Spectral counting which is a strategy to determine a relative quantification of protein from their number of spectra obtained with tryptic peptides in mass spectrometry. This quantification is based on the fact that the more of a particular protein is present in a sample, the more MS spectra are detected for peptides of that protein.

SUPPLEMENTARY FIGURES

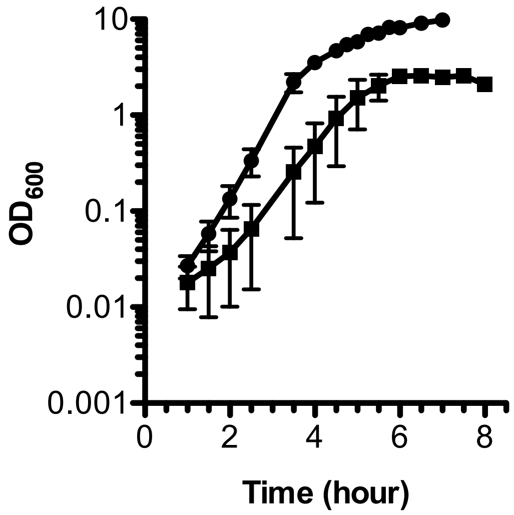

Supplementary Figure 1. Growth curve of Δ*codY* and wild-type cells. Cells were grown in LB at 37°C under agitation. The medium was inoculated with cells grown overnight at 30°C diluted 1000x. Bullets, Bt 407⁻; squares, Bt ΔcodY. The x axis indicates the hours after inoculation of the culture. The results are the mean values of two independent experiments, and the error bars represent standard deviations.

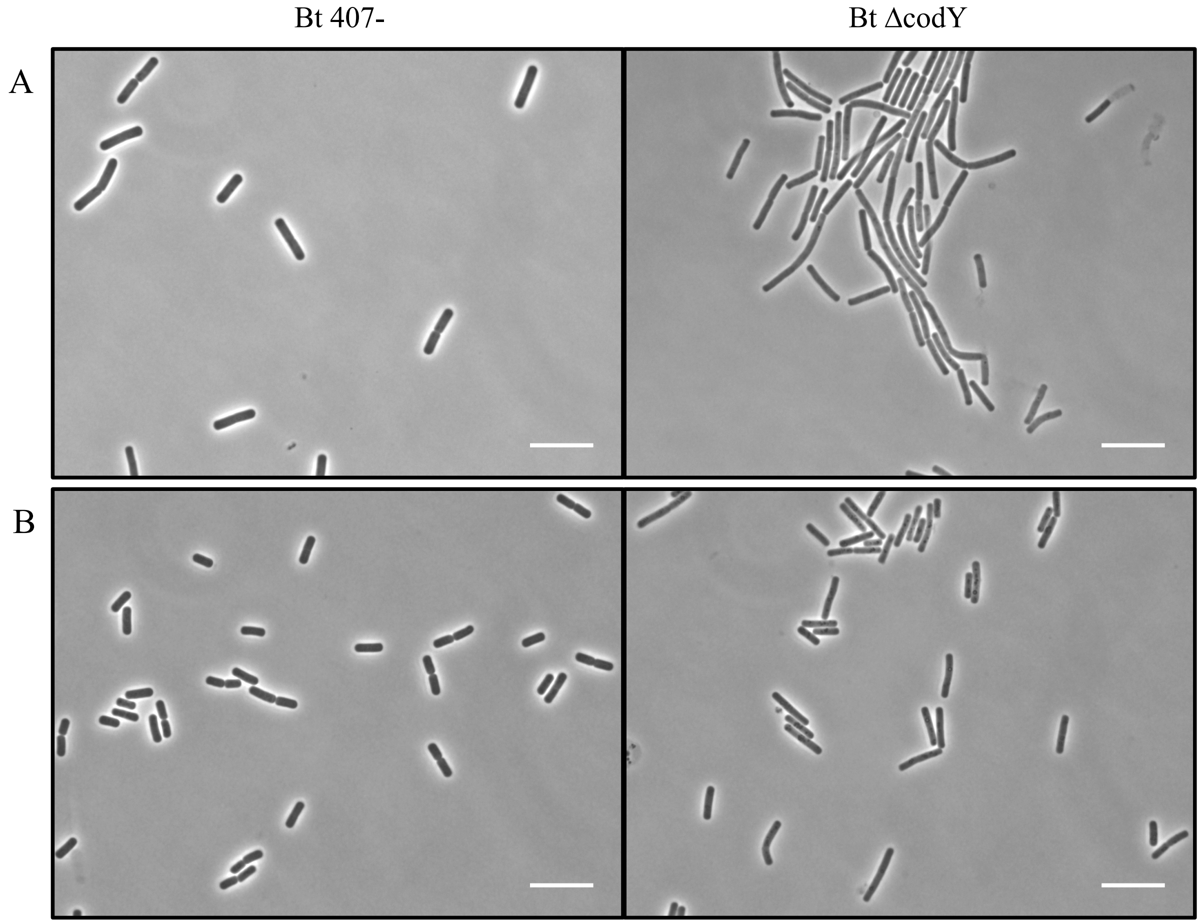

Supplementary Figure 2. Phase-contrast micrographes of Δ*codY* and wild-type cells. Cells were grown in LB at 37°C under agitation and sample were taken 2h before (A) and after (B) the onset of stationary phase. The scale bars represent 10µm.

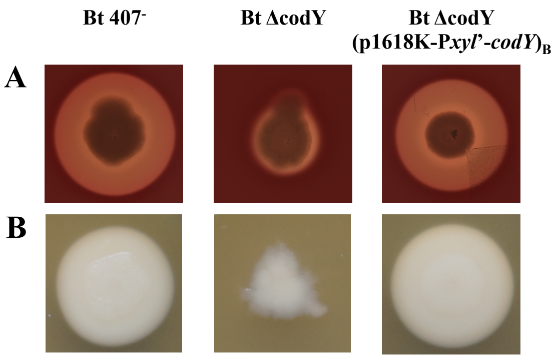

Supplementary Figure 3. Hemolytic and lecithinase activities of Bt ΔcodY. The wild-type, Bt ΔcodY and Bt ΔcodY (p1618K-P*xyl'*-*codY*)$_B$ strains were patched on sheep blood and egg yolk agar plates supplemented with xylose and incubated at 37°C for 24h. The pictures are representative of at least three independent experiments.

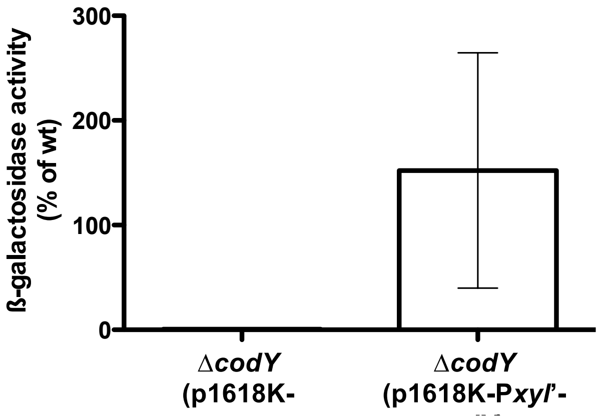

Supplementary Figure 4. Complementation of Bt ΔcodY with an ectopic copy of *codY* restores expression of *plcA*. Strains Bt ΔcodY (pP*plcA*'-*Z*) harboring the p1618K-Pxyl plasmid carrying or not a copy of *codY* under the control of the P*xyl* promoter were grown in liquid LB, supplemented with xylose, at 37°C. Samples were collected 2h after the beginning of the transition into stationary phase (t2) and ß-galactosidase production was assayed. The results are the mean values of two independent experiments, and the error bars represent standard deviations.

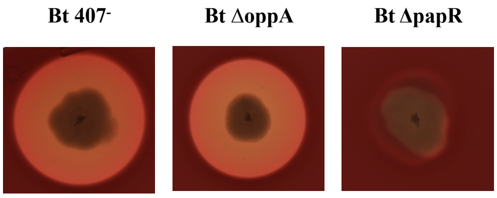

Supplementary Figure 5. Hemolytic activity of Bt ΔoppA. The Bt 407 (p304-P*xyl*+), Bt ΔoppA and Bt ΔpapR P*plcA*'-*lacZ* strains were patched on sheep blood agar plates and incubated at 37°C for 16h. The pictures are representative of at least three independent experiments.

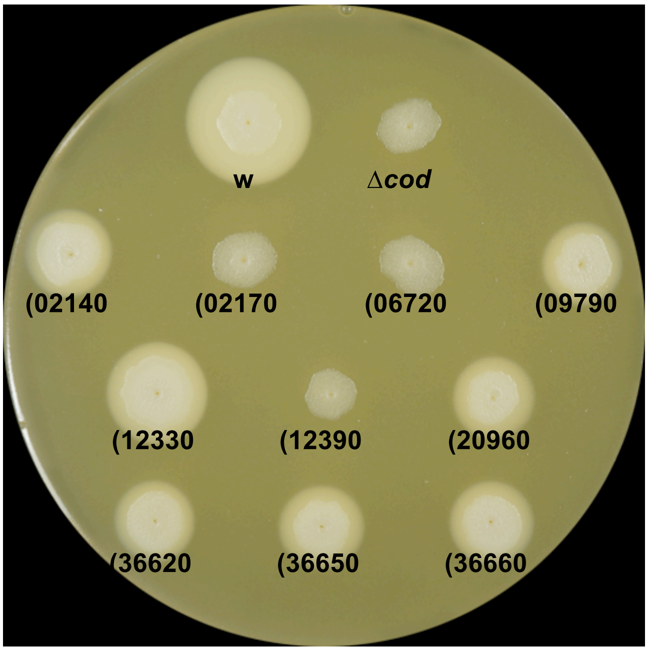

Supplementary Figure 6. Lecithinase activity of Δ*codY* cells over-expressing *oppA* and *oppA*-like-encoding genes

Cells were patched onto egg yolk agar plates supplemented with xylose and incubated at 37°C for 20h. The cells either harbor the empty vector p304-P*xyl*+ or overexpress our gene of interest under the control of a xylose-inducible promoter. wt, Bt 407- (p304-P*xyl*+); Δ*codY*, Bt ΔcodY (p304-P*xyl*+); (02170), Bt ΔcodY (p304-P*xyl*+-02170); (06720), Bt ΔcodY (p304-P*xyl*+-06720); (09790), Bt ΔcodY (p304-P*xyl*+-09790); (12330), Bt ΔcodY (p304-P*xyl*+-12330); (12390), Bt ΔcodY (p304-P*xyl*+-12390); (20960), Bt ΔcodY (p304-P*xyl*+-20960); (36620), Bt ΔcodY (p304-P*xyl*+-36620); (36650), Bt ΔcodY (p304-P*xyl*+-36650); (36660), Bt ΔcodY (p304-P*xyl*+-36660). The pictures are representative of two independent experiments.

SUPPLEMENTARY TABLES

Supplementary Table 1. Primers

Name	5'-3' sequence	Restriction site
codYUp-HindIII	CATGCCATGGAAGCTTGCTGCAGGGCTTCTTGAAGAAGAG	*Hind*III
codYUp-XbaI	GCTCTAGATACGAATACGTTTGCTTCGATT	*Xba*I
codYDn-XbaI	GCTCTAGAGTAGGAATTACTCGTTCTGTAATCGTA	*Xba*I
codYDn-BamHI	CGGGATCCTGTATCAACGATACCGATGATT	*Bam*HI
codY7	GGAGGCGCTCAAAATTCGAATCAAACG	/
codYVerif Rev	ACAGCACGAATTGCATCATCGTTTGCT	/
PR	AGCGGATAACAATTTCACACAGGA	/
xylout	GAAATAAAATGCATCTGTATTTG	/
RN2	CTGGTTTAAGCCGACTGCGC	/
RN3	CACGCCGAAACAAGCGCTCATGAGCCCG	/
Pxyl1	CACATGCATGCCATGTCACTATTGCTTCAG	*Sph*I
PxylRBS+	GCTCTAGACCATGGTGTCACCTCCTTAAAAATAAATTC	*Xba*I
SP1	GGTCTCCCATGCAAGCAGAGAAATTAGG	*Bsa*I
PO2	CGGGATCCAGGTTGTTTATCTGCTG	*Bam*HI
PapR7i1	CATGGCAGATTTACCTTTTGAGTTTTAAGGCGCGCC	/
PapR7i2	AATTGGCGCGCCTTAAAACTCAAAAGGTAAATCTGC	/
PapRFL1	TCGGTCTCTCATGAAGAAATTACTTATTGG	*Bsa*I
PapRFL2	CGGAATTCTTAAAACTCAAAAGGTAAATCTGC	*Eco*RI
Pp3	CCCAAGCTTCCACCTAATGTATCGCCCATAATCC	*Hind*III
Pp2	CGGGATCCTAACAATCTAATTTTTTAAGTTGTC	*Bam*HI
LSoppA1	CGGAATTCGTTTTATAAATACGTTCTTTC	*Eco*RI
LSoppA2	GTAAGTTTTTTCCATCTCACCTGAATACACAAAGTCTTTT GCTG	/
LSoppA3	CAGCAAAAGACTTTGTGTATTCAGGTGAGATGGAAAAAA ACTTAC	/
LSoppA4	CGGGATCCGAAATATTGTAGTAAAGCG	*Bam*HI
VoppA1	CGAGAACAACCCAAGAACG	/
VoppA2	CATTACTGTAAACTCTGGTC	/

c02170.1	GGTCTCTCATGAAGAAAAAAGTTG	*Bsa*I
c02170.2	GGGGTACCTTATTTTGCAACTTCTGTC	*Kpn*I
c06720.1	GGTCTCTCATGAACAAACCAAAACTGT	*Bsa*I
c06720.2	GGGGTACCTTATTTTTCTACTTCTGG	*Kpn*I
c09790.1	GGTCTCTCATGAAGAAGAAGTTGTTTG	*Bsa*I
c09790.2	GGGGTACCTTATTTCACTTCATAAATC	*Kpn*I
c12330.1	GGTCTCTCATGAAGAAAAAGATACCG	*Bsa*I
c12330.2	GGGGTACCTTATTTTTGTTCAACAG	*Kpn*I
c12390.1	GGTCTCTCATGAAAAAAAAGATACC	*Bsa*I
c12390.2	GGGGTACCTTATTTTCCTAGGGATG	*Kpn*I
c20960.1	GGTCTCTCATGAAGAGAAAGACAACAAC	*Bsa*I
c20960.2	GGGGTACCCTATTTTTTTGTAATATATGC	*Kpn*I
c36620.1	GTCACTTCATGAAGAAAAAGATGAAAAAGTTTAC	*Bsp*HI
c36620.2	GGGGTACCCTATTTCTCTTCTACATACG	*Kpn*I
c36650.1	GGTCTCTCATGAAGAAAAAGATGAAAAAGTTC	*Bsa*I
c36650.2	GGGGTACCTTATTTCCCTTCCGTTACATAAG	*Kpn*I
c36660.1	GGTCTCTCATGAAAAAGAAAAAAATGAAAAAAC	*Bsa*I
c36660.2	GGGGTACCTTATTTTCCATCTTTTTCAG	*Kpn*I

Supplementary Table 2. Clones screened for *codY* deletion

Strain used for the growth cycles	*codY*::pRN-Δ*codY*	*codY*::pRN-Δ*codY* (p1618K-P*xyl*)	*codY*::pRN-Δ*codY* (p1618K-P*xyl*'-*codY*)
Number of clones patched after the growth cycles	200	200	200
Number of Erm^S clones	196	199	146
Number of clones tested by PCR	30	8	8
Number of Δ*codY* clones	0	0	1

Supplementary Table 3. Clones screened for plasmid loss

Strain used for the growth cycles	Bt Δ*codY* (p1618K-P*xyl'*-*codY*)	Bt (p1618K-P*xyl'*-*codY*)
Number of clones patched after the growth cycles	200	200
Number of Kan^S clones	3	180
% cells without plasmid	1.5%	90%

Supplementary Table 4. Opp-like-encoding genes in Bt 407[-]

Locus tag in Bt 407[a]	Gene name in Bt 407[a]	Predicted function[b]
02100	*dppB*	OppB-like, transport permease protein
02110	*dppC*	OppC-like, transport permease protein
02120	*dppD1*	OppD-like, ATP binding protein
02130	*oppF1*	OppF-like, ATP binding protein
02140	*dppE1*	OppA-like, substrate binding protein
02170*	*dppE2*	OppA-like, substrate binding protein
02180	*dppE3*	OppA-like, substrate binding protein
02460*	*appA1*	OppA-like, substrate binding protein
02470	*appB1*	OppB-like, transport permease protein
02480	*appC1*	OppC-like, transport permease protein
02490	*appD*	OppD-like, ATP binding protein
02500	*appF1*	OppF-like, ATP binding protein
06720*	*oppA1*	OppA-like, substrate binding protein
06730*	*oppC1*	OppC-like, transport permease protein
06740*	*oppB1*	OppB-like, transport permease protein
06750*	*oppF2*	OppF-like, ATP binding protein
06760*	*oppD1*	OppD-like, ATP binding protein
08880	*oppB2*	OppB-like, transport permease protein
08890	*oppC2*	OppC-like, transport permease protein
09270	*oppA2*	OppA-like, substrate binding protein
09790*	*appA2*	OppA-like, substrate binding protein
09800	*appB2*	OppB-like, transport permease protein
09810	*appC2*	OppC-like, transport permease protein
09820	*dppD2*	OppD-like, ATP binding protein
09830	*appF2*	OppF-like, ATP binding protein
12330*	*dppE4*	OppA-like, substrate binding protein
12340*	*oppB3*	OppB-like, transport permease protein
12350*	*oppC3*	OppC-like, transport permease protein
12360*	*oppD2*	OppD-like, ATP binding protein
12370*	*oppF3*	OppF-like, ATP binding protein
12390*	*dppE5*	OppA-like, substrate binding protein
18470	*dppE6*	OppA-like, substrate binding protein
20960*	*dppE7*	OppA-like, substrate binding protein
24060	*oppB4*	OppB-like, transport permease protein
24070	*oppC4*	OppC-like, transport permease protein
29680*	*dppE8*	OppA-like, substrate binding protein
29990	*oppA3*	OppA-like, substrate binding protein
36620*	*dppE9*	OppA-like, substrate binding protein
36650*	*dppE10*	OppA-like, substrate binding protein
36660*	*dppE11*	OppA-like, substrate binding protein

a As annotated by Sheppard *et al.*, 2013. For ease of reading we removed "BTB_c" from the locus tag and only kept the number associated.
b Using BLAST (http://blast.ncbi.nlm.nih.gov/Blast.cgi)
* Locus tags corresponding to proteins detected in our analysis.

Substrate-binding proteins are indicated in pink. Transport permease proteins are indicated in blue. ATP-binding proteins are indicated in green.
Orphan substrate-binding proteins are indicated by an orange background.
Putative operons encoding all components of a permease are indicated by grey backgrounds.

Supplementary Table 5. Identification of the oligopeptide permease components in membrane-enriched preparations of wild-type and Δ*codY* cells using mass spectrometry

				t-1			t0			t1		
**Locus tag in Bt 407 (old annotation)[a]**	**Locus tag in Bt 407 (new annotation)[b]**	**Gene name in Bt 407[a]**	**Uniprot ID[c]**	**Mean spectra number (wt sample)[d]**	**Mean spectra number (Δ*codY* sample)[d]**	**P value[e]**	**Mean spectra number (wt sample)[d]**	**Mean spectra number (Δ*codY* sample)[d]**	**P value[e]**	**Mean spectra number (wt sample)[d]**	**Mean spectra number (Δ*codY* sample)[d]**	**P value[e]**
**02170**	01090	*dppE2*	C3CCZ0	0.67	6.00	7.18E-03	0.33	8.00	3.10E-03	1.00	1.00	NS
**02460**	01215	*appA1*	K4LPY0	4.67	0.00	NS	5.00	0.00	ND	1.33	0.00	NS
**06720**	03335	*oppA1*	C3CE31	48.33	42.00	NS	44.00	3.00	5.91E-05	61.33	0.67	1.30E-03
**06730**	03340	*oppC1*	C3CE32	4.67	3.67	NS	5.67	0.00	7.02E-05	7.00	0.00	2.66E-04
**06740**	03345	*oppB1*	K4LTM2	4.00	2.00	NS	4.33	0.00	NS	5.33	0.00	1.32E-03
**06750**	03350	*oppF2*	K4LQI2	16.00	8.33	NS	16.33	0.00	3.57E-04	23.67	0.00	8.31E-05
**06760**	03355	*oppD1*	C3CE35	8.33	5.33	NS	10.67	0.00	2.68E-04	17.33	0.00	1.30E-05
**09790**	04865	*appA2*	C3CEV9	3.00	2.00	NS	3.33	3.67	NS	3.67	2.33	NS
**12330**	06075	*dppE4*	J3QW88	0.00	6.00	4.84E-04	2.67	6.67	NS	11.33	0.33	1.80E-03
**12340**	06080	*oppB3*	J3QW74	8.00	7.00	NS	6.33	2.33	NS	11.00	0.00	6.78E-04
**12350**	06085	*oppC3*[f]	J3QW96	14.33	9.67	NS	14.33	5.00	3.65E-02	16.67	0.33	2.87E-04
**12360**	06090	*oppD2*	J3QW75	26.00	22.33	NS	24.33	13.33	1.27E-02	36.67	1.33	4.78E-05
**12370**	06095	*oppF3*	K4LV53	29.33	27.00	NS	32.67	13.00	2.67E-03	42.67	4.67	1.25E-03
**12390**	06105	*dppE5*	K4LV05	0.00	35.33	7.78E-04	0.00	37.33	3.83E-04	1.33	6.67	8.50E-03
**20960**	10315	*dppE7*	K4LU95	48.67	1.67	1.09E-04	45.00	0.00	2.31E-05	58.67	0.00	4.24E-04
**29680**	14560	*dppE8*	C3CJV9	2.00	0.00	NS	0.33	0.00	NS	0.00	0.00	ND
**36620**	17975	*dppE9*	C3CLU3	47.33	8.33	1.27E-03	50.67	0.00	5.50E-07	76.00	0.00	1.04E-04
**36650**	17990	*dppE10*	C3CLU4	57.33	5.67	1.03E-04	59.33	0.00	1.32E-04	81.67	0.00	9.89E-05
**36660**	17995	*dppE11*	C3CLU5	57.33	31.00	9.67E-02	64.67	8.00	2.64E-04	100.67	1.00	9.53E-05

a As annotated by (Sheppard *et al.*, 2013). For ease of reading we removed "BTB_c" from the locus tag and only kept the number associated.

b As re-annotated on the NCBI database (http://www.ncbi.nlm.nih.gov/nuccore/NC_018877.1) (NCBI genome reference sequence NC_018877.1). For ease of reading we removed “BTB_RS” from the locus tag and only kept the number associated.
c As annotated in the Uniprot database (http://www.uniprot.org/)
d The threshold for being referenced in the table was a mean number of spectra of at least 2 in at least 1 condition for the wild-type or the Δ*codY* samples
e As determined using a Student t test
f Manually annotated
ND Not determined. Student t test P value could not be calculated
NS No significant difference in the number of spectra between the wild-type and Δ*codY* samples (difference in spectra <5 and P value ≥0.05)

SUPPLEMENTARY REFERENCES

Sheppard, A.E., Poehlein, A., Rosenstiel, P., Liesegang, H., and Schulenburg, H. (2013). Complete Genome Sequence of *Bacillus thuringiensis* Strain 407 Cry. *Genome Announc* 1. doi: 10.1128/genomeA.00158-12.

Washburn, M.P., Wolters, D., and Yates, J.R., 3rd (2001). Large-scale analysis of the yeast proteome by multidimensional protein identification technology. *Nat Biotechnol* 19**,** 242-247. doi: 10.1038/85686.
